# Circulating Tumor Cells Shed Shearosome Extracellular Vesicles in Capillary Bifurcations That Activate Endothelial and Immune Cells

**DOI:** 10.1002/advs.202506339

**Published:** 2025-06-09

**Authors:** Angelos Vrynas, Salime Bazban‐Shotorbani, Sara Arfan, Karishma Satia, Brian Cunningham, Gauhar Sagindykova, Mymuna Ashna, Aoyu Zhang, Diana Visan, Aisher Chen, Teige Matthews‐Palmer, Mathew Carter, Fiona Blackhall, Kathryn L. Simpson, Caroline Dive, Paul Huang, Sam H. Au

**Affiliations:** ^1^ Department of Bioengineering Imperial College London London SW7 2AZ United Kingdom; ^2^ Division of Cancer Biology The Institute of Cancer Research London SM2 5NG United Kingdom; ^3^ Cancer Research UK National Biomarker Centre University of Manchester Manchester M13 9PL United Kingdom; ^4^ Cancer Research UK Lung Cancer Centre of Excellence Manchester M13 9PL United Kingdom; ^5^ SCLC Biology Group Cancer Research UK Manchester Institute University of Manchester Manchester M20 4BX United Kingdom; ^6^ Division of Structural Biology The Institute of Cancer Research London SW7 3RP United Kingdom; ^7^ Medical Oncology Christie Hospital National Health Service Foundation Trust Manchester M20 4BX United Kingdom; ^8^ The Division of Cancer Sciences Faculty of Biology Medicine, and Health University of Manchester Manchester M13 9PL United Kingdom; ^9^ Cancer Research UK Convergence Science Centre London SW7 2AZ United Kingdom

**Keywords:** cancer metastasis, circulating tumor cells, large extracellular vesicles, premetastatic niche, microfluidics

## Abstract

Circulating tumor cells (CTCs) and their clusters are the cellular drivers of metastasis. This study uses microfluidic models mimicking human capillary bifurcations to better understand how these cells interact with capillary beds. Patient CTCs, CTC‐derived explant cells, and numerous cancer cell lines shed nuclei‐free fragments in a cell size‐ and bifurcation‐dependent manner. These shedding events, which reduces cell sizes up to 61%, facilitate CTC transit through bifurcations. The shed fragments are a novel subclass of large extracellular vesicles (LEVs) that we name “shearosomes” based on their requirement of shear stress for their biogenesis, and whose proteome is associated with immune‐related pathways. Shearosomes exhibit functions that are characteristic of previously identified extracellular vesicles (EVs), including cell‐directed internalization by endothelial and immune cells, and intercellular communication capabilities such as disruption of endothelial barrier integrity, polarization of monocytes toward M2 tumor‐promoting macrophages, and mediating interactions between endothelial and immune cells. These findings suggest that CTCs shed shearosomes within capillary beds that affect cells implicated in the metastatic cascade.

## Graphical abstract

Using microfluidic models of capillary networks, this study discovered that fluid shear stresses on circulating tumor cells transiting through capillary‐sized bifurcations shed a novel class of shear‐dependent large extracellular vesicle, “shearosomes”, whose proteome comprises of over 3000 distinct proteins. Shearosomes are readily internalized by both endothelial and immune cells, damage endothelial barrier integrity, and polarize immune cells toward M2 tumor‐promoting phenotypes.

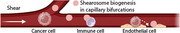

## 1 Introduction

Circulating tumor cells (CTCs) can travel through the vasculature to establish metastatic tumors in distant organs.^[^ ^1^, ^2^, ^3^, ^4^ ^]^ CTCs can become entrapped in capillaries because of the discrepancy in diameters between these vessels (5–10 µm)^[^ ^1^ ^]^ and CTCs, which are often much larger.^[^ ^4^ ^]^ However, entrapment of CTCs alone is not sufficient for successful metastasis. Instead, the microenvironment of distant organs needs to be modified to make these sites more conducive to the migration, survival, and proliferation of disseminated tumor cells.^[^ ^2^, ^3^, ^4^ ^]^ This process, known as the formation of premetastatic niches (PMN), is characterized by the disruption of blood vessel barrier integrity that promotes tumor cell extravasation, the activation of endothelial cells, polarization and/or recruitment of stromal cells including fibroblasts, macrophages, and other immune cells.^[^ ^5^ ^]^ It is currently accepted that primary tumors are responsible for the formation of PMN through the secretion of various signaling molecules and vesicles.^[^ ^5^, ^6^ ^]^ However, we hypothesize that there exists another mechanism whereby CTCs entrapped in capillaries can induce phenotypic changes in stromal cells that alter the metastatic propensities of tumors.

In this work, we characterized the shedding of large cytoplasmic fragments by patient CTCs and CTC‐derived cells entrapped in microfluidic models of human capillary bifurcations. This shedding phenomenon was previously observed in vivo, was attributed to shear stress, and led to the internalization of fragments by monocytes, macrophages, neutrophils, and dendritic cells.^[^ ^7^ ^]^ What we do not understand are the identity of these fragments, the mechanism and frequency of fragment biogenesis, and how these fragments drive key processes in metastasis such as PMN formation. Our analysis indicates that shedding is a biomechanical process that generates a novel class of large extracellular vesicles (LEVs) from CTCs. LEVs were readily internalized by endothelial cells, monocytes, and M1 macrophages and can drive key processes in the formation of the PMN such as disruption of endothelial barrier integrity, endothelial cell activation, polarization of monocytes and M1 macrophages into M2 tumor‐promoting macrophages, and secondary co‐interactions between endothelial cells and immune cells. Overall, we propose that this mechanism of shedding allows CTCs to influence their local microenvironment in ways that improve their probabilities of metastatic success.

## 2 Results

### 2.1 Capillary Bifurcations Promote Cell Shedding of LEVs

**Figure 1:**
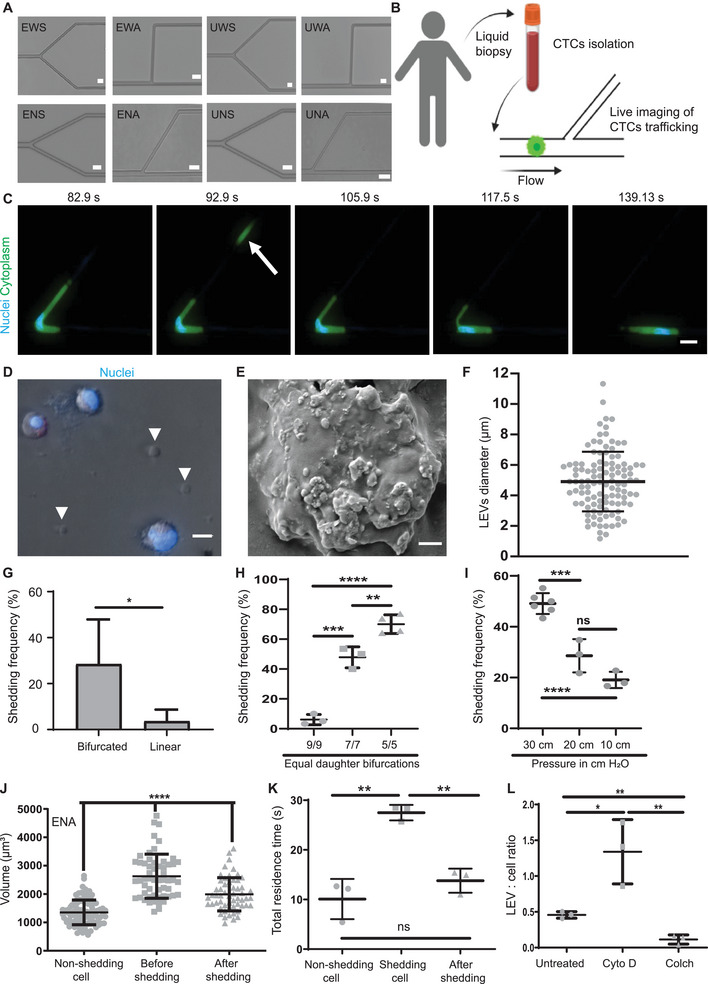
Biogenesis mechanisms of LEVs by tumor cells in capillary geometries and their fate post‐shedding. A) Images of 8 bifurcation variants (E:Equal, U:Unequal, W:Wide, N:Narrow, S:Symmetry, A:Asymmetry). Scale bar 20 µm. B) Experimental workflow of live imaging. Created with Biorender.com. C) Timelapse of a MDA‐MB231 breast cancer cell shedding LEV (white arrow). Cytoplasm was stained with CMFDA cell tracker (green) and nuclei with Hoechst‐33342 (blue). Scale bar 20 µm. D) Hoechst‐positive MDA‐MB231 cells and Hoechst‐negative LEVs. Scale bar 10 µm. E) Scanning electron microscopy image of a LEV. Scale bar 2 µm. F) Diameter (µm) of LEVs (*n* = 111 LEVs). G) Shedding frequency of MDA‐MB231 cells in various bifurcation variants (*n* = 24) versus linear (*n* = 3). Two‐tailed Student's *t*‐test. H) Shedding frequency of MDA‐MB231 cells in ENA_9/9 (*n *= 3), ENA_7/7 (*n* = 3) and ENA_5/5 (*n* = 4). One‐way ANOVA. I) Shedding frequency of MDA‐MB231 cells under various pressures (*n* at least 3). One‐way ANOVA. J) Volume (µm^3^) of MDA‐MB231 non‐shedding cells (*n* = 95), or shedding cells (*n* = 56). One‐way ANOVA. K) Mean total residence time (s) of non‐shedding MDA‐MB231 cells, shedding cells, and after shedding (*n* = 3). Two‐tailed Student's *t*‐test. L) LEV: cell ratio of untreated, Cytochalasin‐D (Cyto‐D) treated, and Colchicine (Colch)‐treated MDA‐MB231 cells, enumerated post‐transit from bifurcation UNA_5/9 (*n* = 3). One‐way ANOVA. All data are presented as mean ± standard deviation (SD). *P*‐values annotated as: * *p* ≤ 0.05, ** *p* ≤ 0.01, *** *p* ≤ 0.001, **** *p* ≤ 0.0001, or not significant (ns).

To mimic the diversity of geometries present in human microvasculature (i.e., capillaries and small arterioles), we engineered 13 distinct variants of microfluidic constricted bifurcation devices alongside constricted non‐bifurcated (linear) (7 µm) and non‐constricted (20 µm) control devices (**Figure** **1**A; Figure S1, Table S1, Supporting Information) alongside computational fluid dynamic simulations of each variant (Figures S2,S3, Supporting Information) that show fluid shear stress profiles of constricted bifurcations within the range of values reported for microvasculature.^[^ ^8^ ^]^ These devices allowed us to systematically explore, via live imaging (Figure 1B), the influence of physiological levels of pressure (10–30 cm H_2_O) on the behavior of primary CTCs isolated from two small cell lung carcinoma (SCLC) patients, SCLC patient CTC‐derived xenograft (CDX) cells, six cancer cell lines (MDA‐MB 231, B16F10, Me290, LNCaP, Panc‐1, OVSAHO), primary cancer‐associated fibroblasts, THP‐1 monocytes, and THP‐1‐derived M0 macrophages at capillary‐sized bifurcations. Upon introduction of cells into microfluidic bifurcation devices, we observed the formation of large (1.17–11.32 µm, mean: 4.9 ± 1.9 µm) Calcein‐AM‐positive, Hoechst‐negative cellular fragments during real‐time transit of all aforementioned cell types at capillary bifurcations within seconds (Figure 1C–F; Figure S4A–F, Movies S1–S4, Supporting Information). We later demonstrate that these fragments matched all the MISEV2018 classification requirements of extracellular vesicles^[^ ^9^ ^]^ (described below), and we henceforth call these fragments “large extracellular vesicles” (LEVs). MDA‐MB 231 cells shed at various constricted bifurcation geometries with a mean frequency of 28.5 ± 19%, a significantly higher rate than in linear geometries (3.6 ± 5%) (Figure 1G) or static conditions (Figure S4G, Supporting Information). Furthermore, centrifugal forces applied to cells at comparable levels of force did not lead to enhanced cell shedding (Figure S4H, Supporting Information). Among the tested geometrical variations in the constricted bifurcation geometries, only smaller daughter bifurcation diameters (Figure 1H) and narrower angles between daughter bifurcations (Figure 4I–L) significantly increased the frequency of cell shedding (*p* < 0.05). As expected, reduction of the pressure across capillary bifurcations led to a decrease in the frequency of cell shedding (Figure 1I). Most cells remained viable after shedding LEVs (described below), including immune cells such as monocytes (Figure S4E, Supporting Information), and the proliferation rate of MDA‐MB‐231 cells post‐transit remained statistically indistinguishable from control cells (Figure S4M,N, Supporting Information). Additionally, clusters of tumor cells also shed LEVs (Figure S5A, Movie S5, Supporting Information), at higher frequencies than single cells (83.8 ± 6% vs 52.2 ± 3%, *p* < 0.05) (Figure S5B, Supporting Information). Clusters that contained three or more cells had higher probability of experiencing at least one shedding event in comparison to doublets (Figure S5C, Supporting Information), and clusters released more LEVs than singlets (Figure S5D,E, Movie S5, Supporting Information), which is likely a reflection of the greater number of cells within clusters.

We then investigated if cytoplasmic fragments that matched the morphological characteristics of LEVs were present in the blood of patients. We first isolated CTCs from blood samples of two SCLC patients (Ethical Committee reference 07/H1014/96) by negative affinity selection and then stained the effluent to identify cellular fragments. We identified 64 and 356 CMFDA‐positive, Hoechst‐negative cellular fragments that matched the morphology of LEVs in these blood samples (Figure S6A,B, Supporting Information). Both patients presented higher counts of cellular fragments compared to CTCs (Figure S6B, Supporting Information), and fragments ranged in size from 1.03 to 8.73 µm (mean: 2.4 ± 1.4 µm) (Figure S6C, Supporting Information).

### 2.2 LEV Biogenesis is a Cell Size‐Dependent and a Volumetric Subtractive Process That Facilitates CTC Transit

We first quantified the total volume of cells before encountering bifurcations in our microfluidic devices. We found that the mean volume of cells that shed LEVs was significantly larger (≈1.9 times) than their non‐shedding counterparts (Movie S6, Supporting Information) during transit in bifurcation variant ENA (2628 ± 8 × 10^2^ µm^3^ vs 1354 ± 4 × 10^2^ µm^3^, *P *< 0.0001) (Figure 1J; Figure S7A,B, Supporting Information) and 1.7–2.4 times larger for the seven other tested bifurcation geometries (Figure S7C–F, Supporting Information). Overall, cell size positively correlated with shedding frequency (*r* ^2^ = 0.61) (Figure S8A, Supporting Information). Smaller cells experienced minimal shedding of LEVs in linear models (Figure S8B, Supporting Information) but were much more capable when transiting through bifurcations (Figures S7C–F,S8C–E, Supporting Information). This size dependency is also supported by our experiments with immune cells, where blood‐resident monocytes shed with significantly lower frequency than larger macrophages (Figure S4D, Supporting Information), which is expected given the larger sizes of macrophages.^[^ ^10^ ^]^ Shedding cells had a mean ≈25% reduction in tumor cell volume post‐shedding during transit in bifurcation variant ENA (2628 ± 8 × 10^23^ vs 1991 ± 6 × 10^2^ µm^3^, *p* < 0.0001) (Figure 1J), which varied from 19 to 22.7% for other tested bifurcation variants (Figure S7D–F, Supporting Information). We also found that bifurcations with equal effective diameter daughter bifurcations caused significantly greater volumetric losses in cells than their unequal counterparts (Figure S9, Supporting Information).

We then recorded how long shedding versus non‐shedding cells remained within capillary bifurcations (residence time). Non‐shedding cells exhibited a mean residence time of 10 ± 4 s (Figure 1K). Cells that eventually shed remained in devices ≈3 times longer (Figure 1K). Interestingly, after shedding LEVs, the mean residence time of cells was dramatically reduced to levels statistically indistinguishable from the residence time of non‐shedding cells (Figure 1K). We postulate that the reductions in cell size imposed by shedding reduced the hydrodynamic resistance of these cells, leading to commensurate reductions in residence times. This suggests that shedding of LEVs facilitates the transit of relatively large but not small CTCs through capillary bifurcations.

### 2.3 F‐Actin Modulates LEV Biogenesis and Post‐Shedding Cell Viability

After exploring the importance of biophysical parameters such as capillary bifurcation geometries and cell size on LEV biogenesis, we wondered whether the actin cytoskeleton was involved in shedding. To this end, we pre‐treated cells with Cytochalasin‐D (Cyto‐D) or Colchicine (Colch) to inhibit or promote F‐actin polymerization, respectively (Figure S10A, Supporting Information) at concentrations that preserved cell viability (Figure S10B,C, Supporting Information). Cyto‐D significantly increased the probability of LEVs biogenesis compared to untreated conditions during single cell transit through capillary bifurcations (Figure 1L; Figure S11A, Supporting Information) or non‐constricted geometries (Figure S11B, Supporting Information). On the other hand, Colch had the opposite effect, reducing LEVs biogenesis in a statistically significant manner compared both to untreated and Cyto‐D treated cells in all tested conditions (Figure 1L; Figure S11B, Supporting Information). Cyto‐D treatment did not significantly increase the shedding frequency from tumor cell clusters in capillary bifurcations (Figure S12A,B, Supporting Information) or non‐constricted geometries (Figure S12C, Supporting Information). We believe that may be because clusters already exhibited high shedding frequency even without chemical treatment (Figure S5B, Supporting Information). Interestingly, Cyto‐D treatment increased LEVs biogenesis from single cells but not clusters under shear (Figure S12D, Supporting Information).

We then quantified cell death 3 h after cell transit in capillary geometries. A small fraction of untreated cells (≈15%) were stained dead after transit in capillary bifurcations (Figure S13A,B, Supporting Information). Interestingly, Cyto‐D‐pre‐treated cells died at statistically significant higher rates post‐transit through bifurcations (Figure S13A,B, Supporting Information) and non‐constricted devices (Figure S13C‐middle, Supporting Information), but remained unaltered in constricted linear geometries (Figure S13C‐left, Supporting Information), static conditions (Figure S13C‐right, Supporting Information) or under Colch treatment (Figure S13B, Supporting Information). Cell viability was further assessed using live cell fluorescent imaging. Lysis of cells during transit in capillary bifurcations was observed (Figure S13D, Movie S7, Supporting Information) and Cyto‐D induced higher rates of cell lysis at capillary bifurcations (Figure S13E, Supporting Information). We then went on to confirm that the higher rates of cell lysis in Cyto‐D‐pre‐treated cells occurred in shedding cells (Figure S13F, Supporting Information), and thus Cyto‐D‐pre‐treated cells had far greater probabilities of cell lysis post‐shedding (Figure S13G, Supporting Information). Altogether, F‐actin modulation influences both LEV biogenesis and viability when cells are subjected to shear and capillary bifurcation forces.

### 2.4 Cytoplasmic Fragments Meet All Established Criteria of LEVs

**Figure 2:**
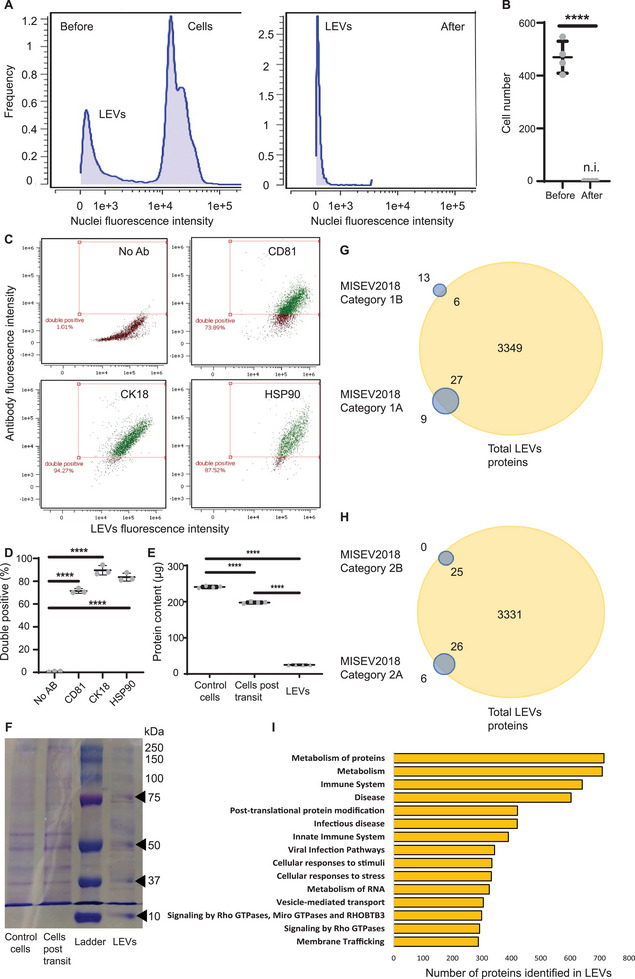
LEVs purification and interrogation of protein content and correlation to downstream functionalities. A) Histogram of MDA‐MB 231 cells and LEVs before and after centrifugation analyzed via flow cytometry. B) Quantification of MDA‐MB 231 cell number before and after centrifugation (*n* = 4). Two‐tailed Student's *t*‐test. C,D) Flow cytometry analysis of LEV proteins. C) Scatter plots of events double positives for CMTPX red cell tracker pre‐stained LEVs and CD81 (top right‐green), CK18 (bottom left‐green), and HSP90 (bottom right‐green). Non‐antibody stained (No Ab) LEVs (top left‐red) were used as control (*n* = 3) and quantification. D) Quantification of data in (C). Two‐tailed Student's *t*‐test. E) Total protein content (µg) of control MDA‐MB 231 cells, cells post‐transit transit and LEVs (*n* = 4). One‐way ANOVA. F) Protein blot for control MDA‐MB 231 cells, cells post‐transit transit and LEVs against a ladder. G) MISEV2018 category 1 transmembrane and GPI‐anchored proteins identified in LEVs through data independent acquisition (DIA) mass spectrometry (MS)‐based proteomics. H) MISEV2018 category 2 cytosolic proteins identified in LEVs through DIA MS‐based proteomics. I) Top 15 Reactome gene sets represented in LEVs proteome. Gene sets are ranked based on the number of overlapping LEV proteins. All data are presented as mean ± standard deviation (SD). *p*‐values annotated as: * *p* ≤ 0.05, ** *p* ≤ 0.01, *** *p* ≤ 0.001, **** *p *≤ 0.0001.

We developed purification protocols using centrifugation or filtration that successfully separated cells and their shed LEVs to purities above 99.9%, validated by flow cytometry (**Figure** **2**A; Figure S14A–D, Supporting Information) and microscopy (Figure 2B; Figure S14E,F, Supporting Information). We then evaluated purified shear‐derived LEVs using the Minimal Information for Studies of Extracellular Vesicles (MISEV 2018) criteria.^[^ ^9^ ^]^ We used immunocytochemistry to verify that LEVs were positive for both cytosolic (CK18, HSP90) and transmembrane (CD81) proteins, similar to their cells of origin, using flow cytometry (Figure 2C,D; Figure S15, Supporting Information), microscopy (Figure S16A–D, Supporting Information) and western blotting (Figure S16E, Supporting Information). We then used three distinct imaging modalities, fluorescent light microscopy (Figure 1D), scanning electron microscopy (Figure 1E), and cryogenic transmission electron microscopy (cryoEM) (Figure S16F, Supporting Information) to visualize LEVs. Furthermore, cryoEM confirmed that vesicles contained cytosol bound by a lipid bilayer with phosphate leaflet spacing of 4 nm (Figure S16F,G, Supporting Information). Densities typical of membrane‐tethered proteins were abundant on the exterior side of the membrane (Figure S16F,G, Supporting Information).

### 2.5 LEVs are Rich in Proteins Relevant to Immune Responses

We then sought to investigate the content of LEVs. Parental cells lost ≈18% of their total protein content after transit through capillary bifurcations (Figure 2E; Figure S17A,B, Supporting Information). About 10% of the original total protein content in cells was detected in recovered LEVs (Figure 2E; Figure S17A,B, Supporting Information), suggesting that some proteins may be shed to the extracellular space during shedding. Further protein analyses revealed the overlap of proteins between LEVs and cells at various molecular weight bands (Figure 2F). Interestingly, similar to other EVs, we found RNA present in LEVs (Figure S17C, Supporting Information), including several mRNA transcripts (Figure S17D, Supporting Information).

We then undertook mass spectrometry‐based proteomic profiling to determine the protein composition of LEVs. Our analysis defined 3382 proteins within the LEVs, representing 60% of all proteins identified in parental cells from which LEVs were derived (see Table S2, Supporting Information and ProteomeXchange – dataset identifier: PXD050444 for the full list of proteins). When compared with the MISEV2018 panel of proteins for categories 1 and 2^[^ ^9^ ^]^, 33 of 55 transmembrane proteins from MISEV2018 categories 1A and 1B (27 non‐tissue specific and 6 cell/tissue specific)^[^ ^9^ ^]^ (Figure 2G, Table S3, Supporting Information) and 51 of 57 cytosolic proteins from MISEV2018 categories 2A and 2B^[^ ^9^ ^]^ (Figure 2H, Table S4, Supporting Information) were identified in LEVs including CK18, HSP90, and CD81, previously identified by immunocytochemistry (Table S2, Supporting Information). Interrogation of Reactome database of the top 15 gene sets which are composed of LEV proteins, identified a range of different biological pathways including multiple immune‐related pathways (immune system, infectious disease, innate immune system, viral infection pathways), regulation of vesicle transport and trafficking (membrane trafficking, signaling by Rho GTPases and vesicle‐mediated transport) as well as protein metabolism (Figure 2I). Interestingly, we identified proteins such as transforming growth factor beta (TGFβ1) and interleukin‐6 (IL‐6) in LEVs (Table S2, Supporting Information), that are mutually responsible for the activation of endothelial cells and monocytes.^[^ ^11^, ^12^, ^13^, ^14^ ^]^ Our proteomic analysis also identified 66 distinct proteins in the LEVs, annotated for protein secretion function (Table S5, Supporting Information), suggesting that LEVs may retain secretory capabilities from parental cells. These prompted us to conduct additional experiments exploring cell‐LEV interactions.

### 2.6 Endothelial and Immune Cells Actively Internalize LEVs

**Figure 3:**
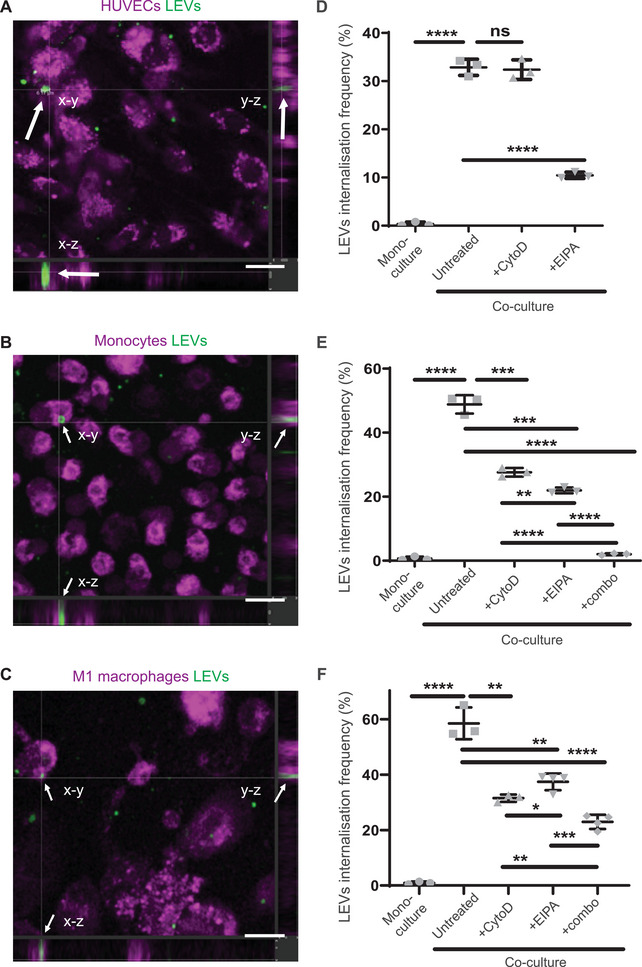
HUVECs, THP‐1 monocytes, and THP‐1 differentiated M1 macrophages internalize LEVs. A–C) Orthogonal cross‐sectioning of multifluorescent confocal images validating the internalization of LEVs by HUVECs (A), monocytes (B), and M1 macrophages (C). Cells were stained with CMTPX cell tracker (shown as magenta) and LEVs with CMFDA cell tracker (green). Scale bar 20 µm. Arrows indicate the internalized LEV in all planes. D–F) Quantification of LEVs internalization frequency (double positive events) by HUVECs (D), monocytes (E), and M1 macrophages (F) (*n* = 3 at least). All data are presented as mean ± standard deviation (SD). *P*‐values annotated as: * *p* ≤ 0.05, ** *p* ≤ 0.01, *** *p* ≤ 0.001, **** *p *≤ 0.0001, or not significant (ns). Two‐tailed Student's *t*‐test was performed for all analyses.

We first explored if LEVs could be actively internalized by cells, similar to previously identified smaller EVs.^[^ ^15^, ^16^ ^]^ We co‐cultured LEVs purified from MBA‐MB‐231 breast cancer cells with human umbilical vein endothelial cells (HUVECs), THP1 human monocytes, or THP‐1 differentiated M1 macrophages. We verified the internalization of LEVs in all tested cell types via co‐localization with the cells’ cytoplasm in Z‐stack slices (**Figure** **3**A–C) and quantified their internalization via flow cytometry (Figure 3D–F; Figure S18, Supporting Information). We also observed that while some LEVs external to cells exhibited chaotic movements consistent with Brownian motion, those co‐localized with cells did not (Movie S8, Supporting Information), further supporting the notion that LEVs were internalized. We also examined if monocytes and M1 macrophages internalized LEVs under flow. Monocytes internalized LEVs under flow to similar levels to static co‐culture conditions (Figure 3E; Figure S19A, Supporting Information), whereas M1 macrophages internalized LEVs under flow to a lesser extent (Figure 3F; Figure S19B, Supporting Information). The mean diameter of LEVs internalized by HUVECs was 2.8 ± 1.2 µm (Figure S20A, Supporting Information), somewhat smaller than the mean diameter of all shed LEVs (4.9 ± 1.9 µm) (Figure 1F).

We then sought to identify the mechanisms responsible for LEV internalization. The two major mechanisms responsible for cellular internalization of large (>1 µm) particles are phagocytosis and micropinocytosis.^[^ ^17^ ^]^ Cytochalasin D, a phagocytosis inhibitor,^[^ ^18^ ^]^ inhibited the mean internalization frequency of LEVs by monocytes by 43% (*p* < 0.0005) (Figure 3E) and M1 macrophages by 46% (*P *< 0.005) (Figure 3F), but not HUVECs (*p *> 0.05) (Figure 3D). On the other hand, 5‐(N‐Ethyl‐N‐isopropyl)‐Amiloride (EIPA), a macropinocytosis inhibitor,^[^ ^18^ ^]^ inhibited the mean internalization frequency of LEVS by monocytes by 55% (*p* < 0.0005) (Figure 3E), M1 macrophages by 36% (*P *< 0.005) (Figure 3F) and HUVECs by 68% (*P *< 0.0001) (Figure 3D). Interestingly, the combination of both Cyto‐D and EIPA fully abrogated LEV internalization by monocytes (Figure 3E), but not entirely in M1 macrophages (Figure 3F). We quantified no internalization of fluorescently labelled beads by any cell type (Figure 20B–D, Supporting Information).

### 2.7 LEVs Polarize Monocytes and M1 Macrophages toward CD206^+^ M2 Macrophage Lineages

**Figure 4:**
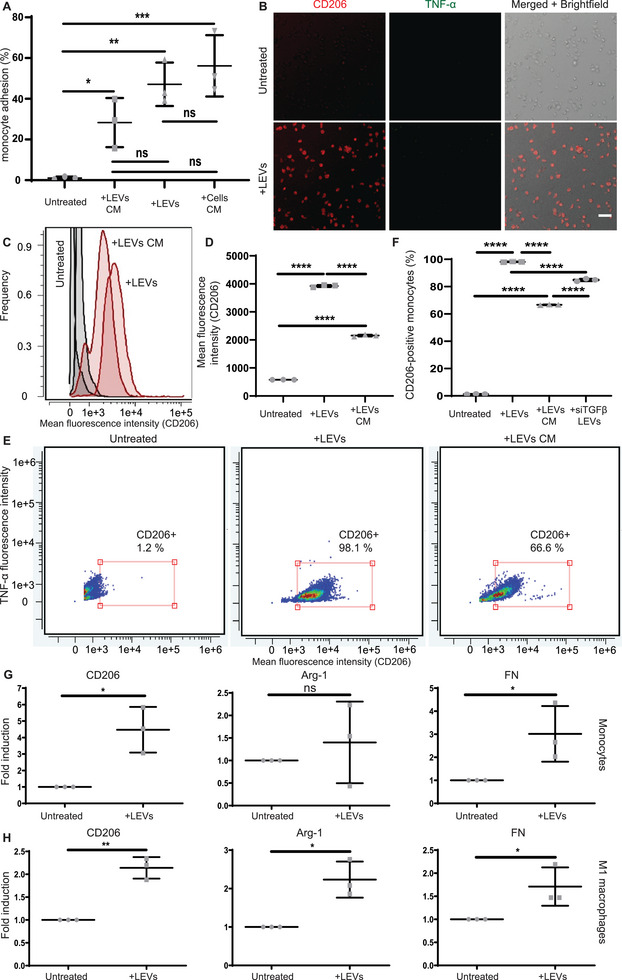
LEVs polarize monocytes and M1 macrophages to CD206^+^ M2 tumor‐promoting macrophages. A) Percentage of monocytes that remained adhered. Monocytes were co‐cultured with LEVs or cultured with CM from LEVs or CM from MDA‐MB231 cells. Untreated monocytes served as a negative control. (*n* = 3). One‐way ANOVA. B) Multifluorescent images of untreated or LEV‐treated monocytes stained for CD206 (red) and TNF‐a (green). Scale bar 50 µm. C,D) Histogram (C) and quantification (D) of CD206 mean fluorescence intensity for untreated monocytes, LEV‐treated monocytes, or monocytes treated with LEVs’ CM for 30 h, analyzed via flow cytometry (*n* = 3). One‐way ANOVA. E,F) Dot plots (E) and quantification (F) of CD206‐positive events for untreated monocytes (left), LEV‐treated monocytes (middle), monocytes treated with LEVs’ CM (right) and monocytes treated with LEVs from siTGFβ‐treated MDA‐MB‐231 cells for 30 h, analyzed via flow cytometry (*n* = 3). One‐way ANOVA. G,H) Reverse transcription quantitative polymerase chain reaction of untreated or LEV‐treated cells for genes CD206 (left), Arginase‐1 (Arg‐1) (middle) and fibronectin (FN) (right), for monocytes (G) and M1 macrophages (H) (*n* = 3). Two‐tailed Student's *t‐*test. All data are presented as mean ± standard deviation (SD). *P* values annotated as: * *p *≤ 0.05, ** *p* ≤ 0.01, *** *p* ≤ 0.001, **** *p* ≤ 0.0001, or not significant (ns).

The polarization of monocytes and macrophage phenotypes is an important contributor to PMN formation.^[^ ^6^, ^19^ ^]^ We found that purified and washed LEVs (details in methods) co‐cultured with THP‐1 monocytes promoted monocyte adhesion (**Figure** **4**A; Figure S21A, Supporting Information), led to the acquisition of stretched morphologies (Figure S21B, Supporting Information) and enhanced proliferation (Figure S21C, Supporting Information). M2 macrophage marker CD206^20^ had higher expression levels in LEV‐treated monocytes (Figure 4B–E), while M1 macrophage marker TNF‐α^[^ ^20^ ^]^ was absent (Figure 4B). To investigate if proteins secreted by LEVs were responsible for monocyte differentiation, we incubated LEVs in cell culture media for 24 h and collected their conditioned media (CM). Monocytes treated with CM from LEVs exhibited significantly lower mean fluorescence intensity of CD206 (Figure 4C,D), and fewer CD206+ cells (Figure 4E,F) than LEV‐treated counterparts. We hypothesized that intravesicular TGF‐β may have contributed to the polarization of monocytes toward M2 macrophages. Silencing siRNA in MDA‐MB‐231 prior to LEV biogenesis led to a ≈20% decrease in the number of monocytes that exhibited polarization markers toward M2 states (Figure 4F). We investigated the degree of M2 macrophage polarization by exploring gene expression levels. Indeed, LEV treatment caused significant upregulation of M2 macrophage‐related genes such as CD206^[^ ^20^, ^21^ ^]^ and fibronectin^[^ ^20^ ^]^ both in LEV‐treated monocytes (Figure 4G) and M1 macrophages (Figure 4H). However, arginase‐1, another M2 marker^[^ ^21^ ^]^ was only statistically increased in LEV‐treated M1 macrophages (Figure 4G,H). Other genes such as interleukin‐6 and CXCL10, whose association to M1 or M2 polarization is contentious, were elevated in some LEV‐treated conditions but not to statistically significant levels (Figure S22, Supporting Information).

### 2.8 LEVs Disrupt the Endothelial Barrier Integrity and Activate Endothelial Cells

**Figure 5:**
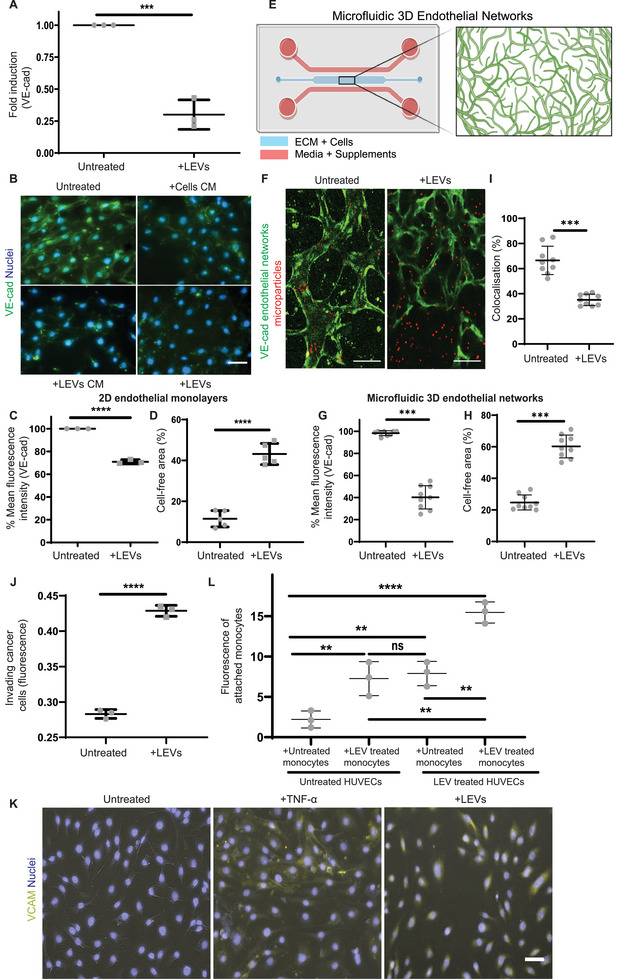
LEVs disrupt and activate the endothelium. A) Reverse transcription polymerase chain reaction of untreated or LEV‐treated HUVECs (30 h) for VE‐cad gene (*n* = 3). Two‐tailed Student's *t*‐test. B) Vascular endothelial cadherin (VE‐cad) (green) staining of LEV‐pre‐treated HUVECs (bottom right), LEVs CM‐pre‐treated HUVECs (bottom left), or MDA‐MB 231 cell CM‐pre‐treated HUVECs (top right) and untreated HUVECs (top left) in 2D. Nuclei were stained with Hoechst‐33342 (blue). Scale bar 50 µm. C) Quantification of VE‐cad % mean fluorescence intensity of HUVECs in 2D, analyzed via flow cytometry (*n* = 3). Two‐tailed Student's *t*‐test. D) Quantification of cell‐free area from untreated or LEV‐treated 2D HUVEC monolayers (*n* = 5 areas). Two‐tailed Student's *t*‐test. E) Schematic of 3D vascular network formation in a microfluidic chip. Created with Biorender. F) Fluorescent staining of microfluidic 3D endothelial networks (green) perfused by dextran 1 µm microparticles (red) in untreated or LEV‐treated microfluidic 3D endothelial networks. Scale bar: 100 µm. G) Quantification of VE‐cad % mean fluorescence intensity of untreated and LEV‐treated 3D endothelial networks (*n* = 3). Two‐tailed Student's *t*‐test. H) Quantification of % cell‐free area of untreated and LEV‐treated 3D endothelial networks (*n* = 3). Two‐tailed Student's *t*‐test. I) Quantification of % of 3D endothelial networks and microparticles colocalization (*n* = 3). Two‐tailed Student's *t*‐test. J) Fluorescence of invading MDA‐MB231 cells after 24 h, in untreated or LEV‐pre‐treated 2D HUVEC monolayers (30 h) (*n* = 3). Two‐tailed Student's *t‐*test. K) Vascular cell adhesion molecule (VCAM‐yellow) staining of untreated HUVECs (top), TNF‐α treated HUVECs (middle), and LEV‐pre‐treated HUVECs (bottom) in 2D. Nuclei were stained with Hoechst‐33342 (blue). Scale bar 50 µm. L) Fluorescence of attached monocytes on 2D HUVEC monolayers (*n* = 3). One‐way ANOVA. All data are presented as mean ± standard deviation (SD). *p*‐values annotated as: * *p* ≤ 0.05, ** *p* ≤ 0.01, *** *p *≤ 0.001, **** *p* ≤ 0.0001, or not significant (ns).

The disruption of the vascular endothelium is an important component of PMN formation.^[^ ^5^, ^6^ ^]^ LEVs added to confluent monolayers of human endothelial cells caused morphological changes (Figure S23A, Supporting Information) and reduced both mRNA (**Figure** **5**A) and protein expression levels of intercellular adhesion molecule VE‐cadherin versus controls (Figure 5B,C; Figure S23B, Supporting Information). Furthermore, LEVs disrupted the continuity of endothelial monolayers, significantly increasing the cell‐free area (Figure 5D; Figure S23C, Supporting Information). Similar to the above experiments, LEVs derived from siTGFβ MDA‐MB231 cells did not lead to reductions in HUVEC cell‐free area, suggesting that intravesicular TGFβ appears to damage endothelial integrity (Figure S23D, Supporting Information). To test whether proteins secreted from LEVs affected endothelial cells, we cultured purified LEVs for 24 h and collected their CM. We found similar disruptions in VE‐cadherin junctions when LEV CM was applied on HUVECs (Figure 5B‐bottom left). To investigate if these findings could be replicated in more physiological microenvironments, we generated 3D endothelial networks in hydrogel‐laden microfluidic devices (Figure 5E). Perfusion of the microfluidic 3D endothelial networks with LEVs (generated as described above) led to similar disruptions to endothelial cells (Figure 5F; Figure S23E, Supporting Information). Treatment with LEVs disrupted VE‐cadherin localization within the 3D endothelial networks, evident by the 60% mean reduction of mean VE‐cadherin fluorescence intensity (Figure 5G). Moreover, analyses of the untreated and LEV‐treated networks revealed that LEV treatment led to an increase in the cell‐free area (Figure 5H), similar to 2D HUVEC monolayers (Figure 5D).

To explore whether these changes in endothelial barrier integrity promoted leakiness, we initially examined the colocalization of fluorescent 1 µm microparticles with microfluidic 3D endothelial networks after perfusion of LEVs (Figure 5F). We quantified a significant decrease in microparticle colocalization with endothelial networks after exposure to LEVs (Figure 5I), suggesting an increase in leakiness. Moreover, we examined the transmigration of MDA‐MB 231 tumor cells and the permeability of fluorescently‐tagged dextran through endothelial cell‐coated Transwell inserts. We noticed a 51% increase in the transmigration of tumor cells (Figure 5J) and a 30% increase in fluorescent dextran permeability through LEV‐treated HUVECs in comparison to controls (Figure S23F,G, Supporting Information).

We then explored if LEVs affected other markers indicative of endothelial activation.^[^ ^22^ ^]^ Compared to untreated controls, LEVs co‐cultured with HUVECs induced significant increases in vascular cell adhesion molecule (VCAM) expression (Figure 5K) and nitric oxide (NO) production (Figure S23H,I, Supporting Information). Since endothelium activation has been correlated to immune modulation via promoting monocyte differentiation^[^ ^23^ ^]^ and attachment to endothelium,^[^ ^24^, ^25^ ^]^ this prompted us to explore how LEVs may mediate the cross‐talk between monocytes and endothelial cells. We therefore cultured untreated or LEV‐pre‐treated HUVECs for 24 h, washed LEVs away, and replenished with fresh media and collected CM from the cells 24 h after. CM from LEV‐pre‐treated HUVECs induced differentiation of monocytes to CD206‐positive, TNF‐α‐negative M2 macrophages, but this was not the case for untreated monocytes or monocytes exposed to CM from untreated HUVECs (Figure S24, Supporting Information). To further examine if LEVs influenced the physical interactions between monocytes and HUVECs, we co‐cultured: a) LEV‐pre‐treated monocytes with HUVECs, b) LEV‐pre‐treated monocytes with LEV‐pre‐treated HUVECs, and c) untreated monocytes with LEV‐pre‐treated HUVECs. We found significant increases in monocyte attachment to HUVEC monolayers when monocytes and/or HUVECs were pre‐treated with LEVs, as opposed to untreated controls (Figure 5L). Altogether, these findings suggest that LEVs shed from entrapped tumor cells drive key aspects involved in PMN formation by disrupting endothelial barrier integrity, activating endothelial cells, and interacting with immune cells.

## 3 Discussion

Our analysis indicates that CTC entrapment in capillary bifurcations can lead to the shedding of a previously uncharacterized class of large extracellular vesicle with the potential to influence key cellular aspects relevant to processes such as PMN formation. We demonstrated that the generation of these particles under physiological microvascular pressures is a shear stress‐driven process that occurs more frequently when: a) cells encounter smaller diameter vessels and bifurcations, b) cells are larger, and c) cells are treated with F‐actin polymerization inhibitors. When the sensitivity of cell shedding of LEVs to capillary‐sized bifurcations, integrity of cytoskeletal elements, and fluid shear stress are taken within the context of the short timescales of particle shedding, we believe that this phenomenon is primarily a biomechanical response that likely effects cells from many solid tumor types. We observed this behavior in all cells that we tested, including patients' CTCs and cells derived from small cell lung cancer, triple‐negative breast cancer, melanoma, prostate cancer, pancreatic ductal adenocarcinoma, or high‐grade serous ovarian cancer.

The large size of fragments shed from tumor cells (1.17–11.32 µm) (Figure 1F) led to reductions of 3–61% in cell volume and facilitated cell transit through capillary bifurcations. The observation that particle shedding may facilitate the transit of CTCs through capillary beds is also supported by previous analyses of CTC hemodynamics by our group and others that show an approximately fifth to sixth power law relationship between cell size and hydrodynamic resistance.^[^ ^26^, ^27^ ^]^ Therefore, even small reductions in cell size may dramatically reduce hydrodynamic resistance and the probability of CTC occlusion.

Reductions in CTC size after transit through capillary bifurcations due to shedding of LEVs may also help to explain previous observations from patient liquid biopsies. The sizes of CTCs isolated from pre‐capillary vessels of metastatic breast cancer (*n* = 30)^[^ ^28^ ^]^ and hepatocellular carcinoma patients (*n* = 73)^[^ ^29^ ^]^ were on average 25–50% larger than those isolated post‐capillary from the peripheral circulation. A likely contributor to this observation is that larger CTCs simply occlude in capillary constrictions and are therefore less abundant downstream of capillary beds.^[^ ^28^ ^]^ However, our data reveals that tumor cells can lose up to 61% (mean: 21 ± 9%) (Figure S7, Supporting Information) of their volume upon shedding, and that shedding can occur in 82% of cells under some conditions. This suggests that forces applied to CTCs in the microvasculature may actively reduce the size of some CTCs via cell shedding, partially explaining the above clinical findings.

We show that fragments shed from CTCs during transit in capillary‐sized bifurcations, match all the criteria of extracellular vesicle classification as outlined by MISEV2018 guidelines, which are: a) imaging of particles using two complementary techniques and b) the demonstration that particles consist of at least three proteins, including at least one transmembrane and one cytosolic protein.^[^ ^9^ ^]^ Given differences in both their mode of biogenesis and their molecular characteristics from previously identified EVs (i.e., exosomes, microvesicles, or large oncosomes (LOs)),^[^ ^9^, ^30^ ^]^ we believe that these cytoplasmic fragments shed from CTCs in circulation are a novel class of large extracellular vesicle. We propose the nomenclature of “shearosomes” for this subclass of large extracellular vesicles because of their unique requirement of shear stress for biogenesis.

Because the diameters of shearosomes (1–11 µm) overlap significant with those of LOs (1–10 µm),^[^ ^30^ ^]^ it is difficult to distinguish these two classes of EVs from patient samples until unique surface markers or biophysical properties can be discovered and validated. Previous proteomic analysis of DU145 prostate cancer cell‐derived LOs identified a total of distinct 407 proteins,^[^ ^31^ ^]^ far fewer than the 3382 distinct proteins we identified in shearosomes from breast cancer cells. We postulate that the greater biomolecule diversity in shearosomes is a direct result of the biomechanical nature of their biogenesis, which differs from previously identified EVs such as exosomes and microvesicles, into which cells actively package biomolecules.^[^ ^30^, ^32^ ^]^ The larger number of proteins present in shearosomes suggests that it is possible to identify numerous shearosome‐specific proteins to separate them from LOs by cross‐referencing the proteomes. The identification of shearosome‐specific markers may allow us to explore both their clinical relevance and compare their influence against other EVs on PMN formation.

A defining characteristic of extracellular vesicles is their uptake by cells.^[^ ^15^, ^16^ ^]^ Our data suggest that endothelial cells utilized macropinocytosis for shearosome uptake, whereas monocytes and macrophages relied both on phagocytosis and macropinocytosis, mechanisms typically associated with the ingestion of particles larger than 1 µm.^[^ ^17^ ^]^ While we observed that the size of generated shearosomes ranged from 1–11 µm, the mean size of ingested shearosomes by endothelial cells was 2.8 ± 1.2 µm (range: 0.93–6.8 µm), suggesting a propensity for uptake of smaller shearosomes. This could be due to inherent size limitations of macropinosome formation, restricting uptake to smaller LEVs. Furthermore, shearosome uptake by endothelial cells or M1 macrophages was not completely abrogated using Cytochalasin D or combo of Cytochalasin D/EIPA inhibitors, respectively. One explanation is that macropinocytosis can occur via different mechanisms, such as lamellipodia‐like protrusions, circular ruffles, and bleb formation.^[^ ^17^ ^]^ It is plausible that endothelial cells utilize more than one of these macropinocytosis mechanisms which the EIPA inhibitor is ineffective against. Moreover, there are various inhibitors used for uptake studies that inhibit at different and sometimes overlapping levels the processes of phagocytosis and macropinocytosis.^[^ ^33^ ^]^ To address this, screening more compounds against the two processes will be necessary to elucidate the complete repertoire of cellular uptake.

The diversity of proteins we identified in shearosomes may have contributed to their profound extracellular communication capabilities and overall reactivity to stromal cells upon uptake. Shearosomes caused: a) the polarization of monocytes and macrophages toward M2 “tumor‐promoting” phenotypes, b) disruption of the endothelial barrier integrity in both 2D and 3D microfluidic models, c) activation of endothelial cells, and d) co‐interactions between these immune and endothelial cells. All these effects are often reported as components in the formation of pre‐metastatic niches,^[^ ^5^, ^6^, ^19^, ^34^ ^]^ which are crucial for successful metastases by regulating CTCs extravasation and eventual colonization.^[^ ^6^, ^7^, ^35^, ^36^, ^37^ ^]^ Shearosomes likely interact with many more components of the complex stromal microenvironment than we explored here. While we found that shearosomes promoted immune cell polarization toward M2 macrophage lineages, Headley et al. found that dendritic cells activated by shearosomes provoked an immune response that appeared to inhibit metastatic burden.^[^ ^7^ ^]^ This raises the question about the overall contribution of shearosomes to malignancy, especially in the context of different immune/stromal landscapes amongst patients and cancer subtypes.

To further explore the contribution of secreted factors from shearosomes, we compared the effects of conditioned media (CM) isolated from shearosomes or direct co‐cultures of shearosomes with endothelial cells or monocytes. CM from shearosomes affected VE‐cadherin expression on endothelial cells and CD206 protein expression on monocytes, albeit at lower levels compared to the effect imposed by co‐cultures with shearosomes. These findings suggest that chemical factors secreted by shearosomes are not solely driving these phenotypic changes and that shearosomes can exert effects on cells also via uptake/binding. These findings are further supported by our proteomic analysis suggesting that shearosomes contain proteins involved in protein secretion, and our experiments using shearosomes generated from TGFβ‐silenced parental cells which had reduced ability to induce both immune cell polarization and endothelial barrier damage.

The extent to which shearosomes contribute to PMN formation remains an open question. Experiments in animal models suggest that a single gram of tumor mass can release ≈4 million cells per day.^[^ ^38^ ^]^ Over the course of a multi‐year disease, primary tumors may therefore release over half a billion cells within four months. Coupled with the frequency of shearosome generation within capillary‐sized bifurcations, it is plausible that endothelial and immune cells interact with appreciable numbers of shed shearosomes. Furthermore, since shearosomes are generated at the site of CTC entrapment, shearosomes may be especially capable of priming the premetastatic niche near microvasculature already enriched for CTCs. While data suggests that shearosomes appear to recruit and modulate immune responses in vivo,^[^ ^7^ ^]^ and to influence key aspects of endothelial and immune cells in manners relevant to metastatic progression, further experiments are needed. Experiments in this area, however, need to be carefully designed, primarily because the biomechanical nature of shearosome biogenesis makes it difficult to devise negative controls where CTCs do not exhibit shedding of shearosomes. For example, while our data suggests that cell treatment with Colchicine inhibits shearosome biogenesis, F‐actin is a key component of the cell cytoskeleton that has crucial roles in cell and tissue homeostasis,^[^ ^39^ ^]^ and therefore more suitable negative controls need to be identified. Moreover, it is unclear how the shedding of shearosomes from CTCs and the production of EVs from primary tumors can compete or synergize toward PMN and eventual metastases formation. More sophisticated in vitro models and carefully designed in vivo studies are therefore needed to investigate the contributions of CTC‐shed shearosomes on components of the pre‐metastatic niche and the progression of metastasis. In conclusion, the shedding of shearosomes by CTCs in the bloodstream appears to facilitate cell navigation through capillary beds and is another driver of phenotypic changes in endothelial and immune cells that may be relevant to cancer progression or metastasis.

## 4 Experimental Section

### CTC Isolation and Enumeration

All reagents were purchased from Sigma Aldrich (UK), unless stated otherwise. Circulating tumor cells (CTCs) were isolated as previously described.^[^ ^40^ ^]^ In brief, 10 mL of EDTA‐treated peripheral blood was collected per patient, from two SCLC patients in December 2023 following informed consent and according to ethically approved protocols. Sample collection was undertaken via the CHEMORES protocol (molecular mechanisms underlying chemotherapy resistance, therapeutic escape, efficacy and toxicity—improving knowledge of treatment resistance in patients with lung cancer), NHS Northwest 9 Research Ethical Committee reference 07/H1014/96) and The National Research Ethics Service, NHS Central Manchester research ethics committee, reference 07/H1008/229.CTCs were enriched via RosetteSep (#15167, Stem Cell Technologies Vancouver, Canada).

CTCs were enumerated as previously described.^[^ ^41^ ^]^ In brief, 7.5 mL blood collected in a Cellsave preservative tube was mixed with 6.5 mL dilution buffer and then centrifuged at 800 × *g* for 10 mins. The sample was loaded onto the AutoPrep where iron beads coated with anti‐Epithelial Cell Adhesion Molecule (EpCAM) antibody (“ferrofluid”) were used to capture candidate circulating rare cells (CRCs) from the sample. Cells that bind the magnetic capture reagent were enriched using a strong magnet which causes them to be drawn to the sides of the tube, the remaining unbound sample was aspirated and disposed. The enriched cells were stained in situ for cytokeratin (CK), CD45 (for the exclusion of lymphocytes), and DNA (DAPI). Enriched cells were deposited in a cartridge which was scanned on the Analyzer, images were presented in a gallery, scored by trained analysts, and the CTC count was reported. Cells were considered to be CTCs if they were DAPI+, CK+, and CD45‐.

Alternatively, during centrifugation to pellet isolated CTCs from SCLC patients, the CTCs pellet was resuspended in 250 µL of media and was introduced directly into microfluidic devices for live imaging of CTCs transit. Additionally, the previously collected supernatant was analyzed to examine the presence of cell fragments. The suspension was initially centrifuged at 150 ×* g* for 7 min (brake applied) to remove any contaminating red blood cells. Then, the supernatant was further centrifuged at 10 000 × *g* for 30 min to pellet potential cell‐derived fragments. The pellet was resuspended in a staining solution containing CMFDA green cell tracker and Hoechst 33342, at final concentrations of 10 and 16.2 µm, respectively, and then imaged in wells of a 96‐well plate using a fluorescent inverted Leica microscope (Leica, UK). Fluorescent fragments that were CMFDA‐positive and Hoechst‐negative, were manually enumerated.

### CTC Derived Explant Generation, Disaggregation, and Culture

CDX models were generated, disaggregated, and cultured as previously described according to UK Home Office Regulations and the UK Coordinating Committee on Cancer Research guidelines using approved protocols (70/8252). In brief, 10 mL of EDTA‐treated peripheral blood was collected from patients with SCLC enrolled in the ChemoRES study (07/H1014/96). CTCs were enriched by means of RosetteSep and subcutaneously implanted into the flank of 8 to 16‐week‐old non‐obese, diabetic, severe combined immunodeficient, interleukin‐2 receptor γ–deficient (NSG) mice (Charles River Laboratories International, Inc., Wilmington, MA). CDX models were generated from the patients’ CTCs enriched from blood samples at pre‐chemotherapy baseline or at post‐treatment disease progression time points.^[^ ^40^, ^42^ ^]^

CDX tumors were grown to ≈800 mm^3^, and the mice were killed by the Schedule 1 method. The tumors were removed and dissociated into single cells using the Miltenyi Biotec tumor dissociation kit (#130‐095‐929 [Miltenyi Biotec, Germany]) following the manufacturer's instructions on a gentleMACS octo dissociator (#130‐096‐427 [Miltenyi Biotec]), as previously described. Single cells were incubated with anti‐mouse anti‐MHC1 antibody (eBioscience clone, 34‐1‐2s [ThermoFisher Scientific, Waltham, MA), anti‐mouse anti–immunoglobulin G (IgG) 2a+b microbeads and dead cell removal microbead set (Miltenyi Biotec #130‐090‐101) and applied to an LS column in a MidiMACS Separator (Miltenyi Biotec) for immunomagnetic depletion of mouse cells and dead cells. CDX ex vivo cultures were maintained in Roswell Park Memorial Institute (RPMI) 1640 medium supplemented with the following components: 10 nm hydrocortisone, 0.005 mg mL^−1^ Insulin, 0.01 mg mL^−1^ transferrin, 10 nm β‐estradiol, and 30 nm sodium selenite; 5 µm Rho kinase inhibitor added fresh (Selleckchem, Y27632 [Houston, TX]), and 2.5% fetal bovine serum added after 1 week at 37 °C and 5% carbon dioxide.^[^ ^40^, ^42^ ^]^

### Cell Culture

Cell lines were obtained from ATCC, unless noted otherwise. MDA‐MB‐231, OVSAHO, LNCaP, Me290, Panc‐1, and B16F10 cell lines were maintained in StableCell‐Dulbecco's Modified Eagles Medium (DMEM) media, supplemented with 10% (v/v) FBS and 1% (v/v) penicillin/streptomycin and incubated at 37 °C/5% CO_2_. Human umbilical vein endothelial cells (HUVECs) were incubated at 37 °C/5%CO_2_ and supplemented in 10% FBS and EGM‐2 Endothelial Cell Growth Medium‐2 BulletKit (Lonza, Switzerland). HUVECs and all other cancer cell lines were sub‐cultured as adherent cells every 2–3 days.

THP‐1 monocytes were maintained in RPMI‐1640, further supplemented with 10% (v/v) heat‐inactivated fetal bovine serum (FBS) and 25 mm 4‐(2‐hydroxyethyl)‐1‐piperazineethanesulfonic acid (HEPEs) buffer (Gibco, US), 2.5 g L^−1^ Glucose (Merck, UK), 1 mm sodium pyruvate (Gibco, US), 1% (v/v) Penicillin/Streptomycin and 50 pm β‐mercaptoethanol (Gibco, US) final concentrations, in suspension. After thawing, THP‐1 monocytes were passaged every 3 days at a 1:4 split ratio. M1 macrophages were obtained as previously described^[^ ^20^ ^]^ by first treating monocytes with 150 nm phorbol‐12‐myristate‐13‐acetate (PMA) for 24 h to obtain M0 macrophages and then further treatment with Interferon gamma (20 ng mL^−1^ IFN‐γ/Biotechne, UK) for 24 h to obtain M1 macrophages.^[^ ^20^ ^]^

Primary cancer‐associated fibroblasts (CAFs) cells were provided by Breast Cancer Now Tissue Bank and were maintained in DMEM: F12, (supplemented with 1% (v/v) penicillin/streptomycin, amphotericin‐B (2.5 µg mL^−1^) and 10% (v/v) heat‐inactivated FBS and incubated at 37 °C/5% CO_2_. Primary CAFs were sub‐cultured every 3 days.

### Preparation of Single Cell and Cluster Suspensions

For single cell experiments, cancer cell lines and primary CAFs (mentioned above) were harvested from 80–90% confluent 48‐well plates (corning, UK) or 25 cm^[^ ^2^ ^]^ tissue culture flasks (Corning, UK), by washing with PBS, followed by incubation with 0.25% trypsin (5 min at 37 °C/5% CO_2_), inactivation with 10% serum‐containing media, centrifugation at 180 × *g* for 5 min and resuspension of the pellet with fresh media in new culture flasks. Alternatively, THP‐1 monocytes (suspension cell line) were collected, centrifuged as above, and enumerated. THP‐1 derived M0 macrophages were harvested as adherent cells and after trypsinization were scraped using cell scrapers. Where required, the cell pellet was resuspended with staining solution for 15 min at 37 °C/5% CO_2_. Staining solutions were prepared to a final volume of 1 mL by diluting to final concentrations of Calcein‐AM (5 µm) and/or Hoechst 33342 (16.2 µm) in PBS as required.

For cell cluster experiments, MDA‐MB 231 cells were initially harvested, as described above, except the pellet was resuspended in FBS‐containing media, and cells were added to round‐bottom 96‐well plates (Corning, UK) for 1 day at 37 °C/5% CO_2_. Round 96‐well plates were pre‐treated with 1% Pluronic F‐127 (Thermo Fischer Scientific, UK) at room temperature for 1 h to facilitate the formation of spheroids. To stain, 200 µL of the solution with both Calcein‐AM and Hoechst (described above) was added directly in the wells. Stained spheroids were gently resuspended to form clusters and suspensions were collected.

### Chemical Treatment of Cells

Single cell and/or cluster suspensions of MDA‐MB 231 cells were pre‐treated with Cytochalasin D (Cyto‐D) (Merck Life Science, UK), or Colchicine (Colch) (Merck Life Science, UK), for 3 h at 37 °C/5% CO_2_, at final working concentrations of 10 µm, 50 µm respectively, as required.

For internalization inhibition studies, HUVECs, THP‐1 monocytes, or THP‐1 derived M1 macrophages were pre‐treated with Cyto‐D or 5‐(N‐Ethyl‐N‐isopropyl)‐Amiloride (EIPA) for 3 h at 37 °C/5% CO_2_, at final working concentrations of 10 and 50 µm, respectively.

To generate positive controls of disrupted HUVEC endothelium, HUVECs were pre‐treated with tumor necrosis factor alpha (ΤNF‐α) at a final working concentration of 0.1 µg mL^−1^ for 30 h.

To evaluate intracellular production of nitric oxide by HUVECs, untreated or LEV co‐cultured HUVECs (elaborated later) were stained with DAF‐FM dye (ThermoFischer Scientific) for 1 h at a final concentration of 5 µm at 37 °C/5% CO_2_.

### Microfluidic Capillary Devices Design, Fabrication, and Preparation

Capillary bifurcations were designed according to Murray's law (Equation (1)):

(1) $${d_{0}}^{3}={d_{1}}^{3}+{d_{2}}^{3}$$

where, *d*_0_, *d*_1_, and *d*_2_ are the effective diameters of the parent and daughter channel vessels in a capillary bifurcation.

To calculate the effective diameter of microchannels narrower than 10 µm, we used Equation (2).

(2) $$d_{e}=1.3*{\left(a*b\right)}^{0.625}/{\left(a+b\right)}^{0.25}$$

where, *a* is the width of the channel and *b* the height.

Equations (1) and (2) were then used to generate 13 distinct capillary bifurcations and two control geometries, as outlined in Table S1 (Supporting Information). Distinct geometries of capillary bifurcations were generated by altering channel dimensions, the symmetry or angle between the two daughter channels, and whether or not the daughter channels had equal or unequal dimensions. These were designed to recapitulate the diversity of bifurcations present in in vivo capillaries.^[^ ^1^ ^]^

All devices were fabricated using photolithography and soft lithography. Briefly, silicon wafers (MSE Supplies, Germany) were sterilized with 99% IPA and 100% acetone and oxygen plasma treated (Harrick plasma cleaner) at 0.5 Tor O_2_ at 30 W for 1 min before spin coating with a GM1050 SU‐8 negative photoresist (Gersteltec, Switzerland). Wafers were spun first at 1050 rpm for 10 s and then at 950 rpm for 45 s, and then heated at 65 and 95 °C for 2 and 10 min, respectively. SU‐8 coated wafers were exposed to UV photolithography with a UV‐KUB 3 mask aligner (KLOE, France) to pattern the capillary designs for all masters, using chromium glass masks (Microlitho, UK). After repeating the heating steps from earlier, wafers were developed in SU‐8 developer (Kayaku Advanced Materials, USA) for 5 min. The height of the devices was set to 7 µm for constricted capillaries and 20 µm for non‐constricted geometries and was verified, using an optical surface profilometer (FILMETRICS, USA), within a ±5% range of intended height. The master wafer was taped in a petri dish and Sylgard 184 Polydimethylsiloxane prepolymer with its crosslinker (Dow Corning) was mixed at a 10:1 w/w ratio, poured onto silicon master molds, degassed for 30 min, and transferred in an oven, left to polymerize overnight at 65 °C.

Polymerized PDMS replicas were cut out, punched in their inlets and outlets with 4‐ and 2‐mm biopsy punch (IHC WORLD, USA), respectively, and then plasma treated with oxygen (0.5 mm Torr, 30 W, 1 min/Blackholelab, France), bonded at glass slides and heated at hotplate for 10 min at 95 °C. The formed microfluidic devices were flushed 1× with 70% v/v ethanol to sterilize and wet the devices, washed 1× with PBS and 3% Bovine Serum Albumin to prevent cell attachment within the devices. To perform cell experiments, 80‐cm long sections of 2 mm outer diameter tubing (Qosina, USA) were connected to 10‐mL polypropylene syringes, coupled with 22‐gauge needles (Needlez, UK). The tubing/syringe set‐up was prefilled with 2 mL PBS, the plungers were removed, and the free ends of the tubing were connected to the outlets of the devices.

### Computational Fluid Dynamics

Microfluidic channel geometries were imported from AutoCAD into ANSYS 2025 R1 Discovery. Meshing was conducted using ANSYS Meshing using quad‐dominant sheet body structured meshes of element sizes of 1 µm. No slip boundary conditions were used at walls, and gauge pressure of the inlets was set at 1961 Pa for all except for non‐constricted bifurcations which were set at 490 Pa. ANSYS Fluent pressure‐based solver was used with water (density = 998 kg m^−^^3^, viscosity = 0.001 Pa s). Coupled solver scheme, second‐order pressure, and second‐order upwind momentum spatial discretization. Convergence criteria set to 1 × 10⁻5 for continuity and momentum equations, and 200 iterations. Fluid shear stress was calculated as the product of strain rate and molecular viscosity.

### Capillary Transit Experiments

Thirty to fifty microliters of single cell suspensions of patient CTCs or 0.2 × 10^6 ^cells mL^−1^ of CDX cells, cancer cell lines, primary CAFs, THP‐1 monocytes and THP‐1 derived M0 macrophages (as mentioned above) were added in the inlets of microfluidic devices, whose outlets were connected to the tubing/syringe set‐up. The syringe was lowered 10–30 cm below the level of the device for constricted bifurcations and 5–10 cm below for non‐constricted bifurcations to generate physiological pressures^[^ ^43^ ^]^ and the cells were collected from the outlets of the devices and by emptying the liquid content from the tubing into a 15 mL falcon tube. Similarly, cell transit was tested both in 10 and 20 cm hydrostatic pressure to investigate the impact of various shear stress values on cancer cell shedding. Where required, cell transit through the microfluidic devices (*n* = 3 per geometry) was imaged using Nikon Eclipse Ti2 multi‐fluorescent inverted microscope (Nikon, UK) with Okolab incubated stage (Nikon, UK). For no‐flow control experiments, cells were added in the inlets of microfluidic devices (*n *= 3) but without the implementation of flow, for the same amount of time that the microfluidic transit experiments lasted before extraction from device inlets. As additional no‐flow control experiments, MDA‐MB 231 cells were stained as previously and transferred in separate Eppendorf tubes, subjected to centrifugation forces (8000 × *g*) for 3 min, transferred to separate wells, and the LEV: cell ratio was calculated. Cells without centrifugation were also analyzed as a control. When required, cells were pre‐treated with Cyto‐D or Colch, as described above.

### Image and Video Analysis

The analysis of individual cells and clusters transiting through capillary bifurcations and control devices was conducted in NIS‐Elements (Nikon, UK). As described above/below, cell shedding was verified by detachment of cytoplasmic‐stained fragment, but negative for nuclei (Hoechst). Shedding frequency for single cell experiments was calculated by dividing the number of cells that shed at least one LEV by to the overall number of cells that transited through a capillary bifurcation. For clusters, shedding frequency was calculated by dividing the number of clusters that shed at least once to the overall number of clusters that transited.

The spherical diameters of cytoplasmic or nuclear volumes were calculated as previously described.^[^ ^26^ ^]^ Briefly, from video frames of the cells within microchannels, cells were constricted only in the vertical axis. Alternatively, the volume of cells trapped in the capillary bifurcation was calculated by a method similar to what was previously described.^[^ ^26^ ^]^

Cell lysis during transit in capillary devices was quantified by examining cells that experienced loss of Calcein‐AM localization. The lysis frequency of shedding cells was extrapolated by dividing the number of cells that were lysed post‐shedding to the number of all cells that shed.

### Viability Assay

One hundred microliters of cell suspensions (10^5^ cells mL^−1^) were added in each well of a 96‐well plate and left to attach and grow for a day. Next, cells were treated with Cyto‐D or Colch, as described above, for either 3 or 24 h at 37 °C/5% CO_2_. Viability of cells at 0 h was also examined as a control. The media was removed at the relevant timeframe, and the staining dye for the live/dead assay was added for 15 min at 37 °C/5% CO_2_. A staining solution consisting of 1× Propidium Iodide (Thermofischer Scientific, UK), 5 µm Calcein‐AM (5 µm), and 16.2 µm Hoechst 33342 in 1 mL media was added to cells. For each condition, 4–5 multifluorescent images were obtained at 20× magnification using the Nikon microscope. Alive cells were defined as Calcein^+^, Hoechst^+^, and Propidium Iodide^−^, whereas dead cells as Calcein^−^, Hoechst^+^, and Propidium Iodide^+^. Viability was assessed by extrapolating the percentage of alive cells compared to the overall number of cells (Hoechst‐positive). Images were analyzed using the Nikon software.

### Post Transit Cell Analysis

Fifty microliters of suspensions of cells and their shed LEVs were collected into wells of 96‐well plates as described above. The diameters of LEVs (Hoechst 33542‐negative, Calcein‐AM positive) were measured using both NIS‐Elements (manually measuring diameters using the Annotations and Measurements command) and automated Cell Profiler scripts. In addition, fluorescent images of LEVs (positive for green cell tracker) and cells (positive for green cell tracker and Hoechst) were obtained and analyzed via CellProfiler scripts to measure the diameter of LEVs. In particular, the workflow consisted of i) uploading of raw fluorescent images, ii) identification of objects (both LEVs and cells), iii) separation of cells from LEVs that are negative for Hoechst (blue signal), and iv) measurement of object diameter. Cell death post‐transit was assessed by performing a viability assay as elaborated above 3 h after transit of either MDA‐MB 231 or THP‐1 monocytes and calculating the percentage of dead cells per condition (*n* = 3). LEVs and cells per condition were enumerated manually (*n* = 3), by analyzing multiple representative multifluorescent images across each well, thus establishing a LEV: cell number ratio.

### Electron Microscopy

Cells and their shed LEVs were collected as described above, and the solution was centrifuged at 180 × *g* for 5 min to separate cells (pellet) and retain LEVs in the supernatant (described below in more detail).

For cryogenic Transmission Electron microscopy, 3 µL of concentrated LEV suspension was applied by pipette to glow‐discharged Quantifoil R2/2 carbon support films on 200‐mesh EM grids. The grids were blotted and vitrified by plunge‐freezing in ethane using an FEI Vitrobot MkIV with a chamber at 22 °C and 100% humidity. Grids were imaged at 200 keV on an FEI Glacios TEM under cryogenic conditions. Overview images for LEV characterization and diameter measurement were collected at 50 µm underfocus at a range of low magnifications. High magnification imaging to characterize the LEV boundaries was performed using a Falcon4i direct electron detector (Thermo Fisher Scientific) and zero‐loss‐centred energy filtering at 10 eV slit width using a Selectris energy filter (Thermo Fisher Scientific). Exposures were collected of 40 e^−^ Å^−1[^ ^2^ ^]^ total dose at 1.18 Å pixel size and 3µm underfocus, generating movies that were aligned and summed in Thermo Fisher's Velox software. Measurements on images were made manually using IMOD.

For Scanning Electron microscopy, LEVs were transferred onto 10 × 10 mm square glass slides that were positioned into wells of a 24 well plate. After 1 day, the media was removed, and the glass slides were fixed with 4% formaldehyde (diluted in PBS) for 15 min at room temperature to crosslink proteins. Then, glass slides were chemically treated with 1% (v/v) Osmium tetroxide (Agar Scientific, UK) to crosslink lipids. The fixed samples were dehydrated with a sequence of washes with increasing concentrations of ethanol, starting from 30% and continuing with 40%, 50%, 60%, 70%, 80%, 90%, and 100% (v/v) ethanol. After dehydration, the samples were chemically dried by using Hexamethyldisilazane (Merck, UK) for 3 mins. A sticky carbon tape was attached to each square glass slide, and silver‐containing glue was brushed onto the samples to enhance conductivity. Finally, a 15 µm thick layer of Nickel was deposited onto the samples using a sample sputter coater (Q150TS, Quorum technologies, UK), to improve image resolution. Prepared samples were loaded into the stage holder of the scanning electron microscope (Zeiss Auriga 40 Cross beam scanning electron microscope, Zeiss, Germany). Acceleration voltage (5 kV; using secondary electron detection) was applied to the sample. Samples were scanned to identify LEVs and images at 1000–8000× magnification.

### LEV Generation, Isolation, and Purification

Two milliliters of untreated MDA‐MB 231 cell suspensions (10^6^ cells mL^−1^) were pre‐stained with a CMTPX red or CMFDA green cell tracker (ThermoFischer Scientific, UK) for 1 h at a final concentration of 5 µm. Then, 100 µL of the suspension was added in the inlet of the capillary bifurcation device (ENA_7/7) (Table S1, Supporting Information) and operated as described above. Before the inlet emptied completely, an additional 100 µL of the suspension was added sequentially until the entire 2 mL volume passed through the device. Both cells and shed LEVs were collected as described above. Two distinct methodologies were used to purify LEVs from cells in effluent solutions. In the first method, filtration, cells and LEVs containing suspensions were transferred to 10 mL syringes and passed through Whatman Polydisc filters with a pore diameter of 10 µm which permitted LEVs to pass through. In the second method, centrifugation, the cell/LEVs suspensions were centrifuged for 5 min at 180 ×* g*, separating cells into the pellet from the LEVs in the supernatant. Supernatants were collected and further centrifuged for 30 min at 10 000 × *g* to form LEV pellets which were later resuspended in fresh media.

To evaluate LEV purity after filtration, 100 µL of the samples before and after filtration were transferred into separate wells of a 96‐well plate, and multifluorescent images of each well were taken as described above. After centrifugation, LEV purity was evaluated by flow cytometry (described below) of 500 µL samples taken before centrifugation or in the pellet and supernatant after centrifugation in an Amnis CellStream flow cytometer (Luminex, US).

### Post Transit Cell Proliferation

To evaluate the proliferative capabilities of MDA‐MB231 cells after transit, 5 × 10^5^ cells were flowed through a microfluidic device, the effluent collected, and cells separated from LEVs as described above. Cells were enumerated using a hemocytometer (as above). Two‐fifty microliters of cell suspension of 0.5 × 10^6 ^cells mL^−1^ density were added to wells of a 48‐well plate. Equal numbers of cells that did not transit through the devices were added to separate wells as controls. Images were taken 4 h after seeding and then every day up to 7 days post‐seeding. In other experiments, post‐transit cells and control cells were left to grow for 3 days in 48‐well plates and were then harvested and enumerated using a hemocytometer.

### Co‐Culture of Cells with LEVs or Conditioned Media

HUVECs, THP1 monocytes, or THP1‐derived M1 macrophages were independently stained with a CMTPX red cell tracker and were independently co‐cultured with MDA‐MB‐231 derived LEVs pre‐stained with CMFDA green cell tracker (as prepared elsewhere) at cell: LEV ratios of 3:1. For LEV internalization studies, co‐culture was allowed to proceed for 16 h. For all other studies, the co‐culture was performed for 30 h to allow for phenotypic changes and gene expression changes.

Conditioned media (CM) was collected from the cell culture supernatant of MDA‐MB 231 cells or HUVEC cells cultured for 1 day (as described above). Cell culture supernatant was centrifuged at 180 × *g* for 5 min, and the supernatant was collected as CM. To collect CM from co‐cultured HUVEC cultures, HUVECs were first co‐cultured with LEVs for 30 h. Media was removed from each well to remove non‐internalized LEVs, and fresh HUVEC media (elsewhere) was added. After 1 day of culture at 37 °C/5% CO_2_, the media was collected and processed as above to collect CM from LEV‐pre‐treated HUVECs. CM from LEVs was collected by purifying and centrifuging LEVs as above, resuspend them in fresh media, culture them for at least 24 h at 37 °C/5% CO_2_ and centrifuge again at 10 000 × *g* for 30 min to collect supernatant, designated as LEVs‐CM.

### Confocal Microscopy

HUVECs, monocytes, or M1 macrophages were co‐cultured as described above with LEVs. Cells were pre‐stained with cell tracker red (CMTPX) (adjusted to magenta) and LEVs with cell tracker green (CMFDA). After 16 h of co‐culture, 96‐well plates were loaded in the stage of a Leica SP8 inverted scanning confocal microscope (Leica, UK). Images were obtained using a Z step of 2 µm. Excitation and emission wavelengths for cells and LEVs were 561/611 and 488/528 nm, respectively. Internalization of LEVs by cells was validated by applying orthogonal cross‐sectioning to image a cell with an LEV in all pairs of coordinates, that is, *x–y*, *x–z*, and *y–z*. Moreover, the diameter of LEVs that were internalized by HUVECs was measured, after first verifying LEVs' internalization.

### Immunocytochemistry

A standard protocol was adopted for immunocytochemistry (ICC) staining. Cells or MDA‐MB 231 derived LEVs were fixed with 4% (v/v) formaldehyde at room temperature for 15 min. Then, cells or LEVs were washed 3× with PBS for 3 min (per wash), incubated with 0.1% (v/v) Triton X at room temperature for 5 min, washed 3× with PBS for 3 min (per wash) and finally incubated with the primary antibody overnight at 4 °C. After overnight staining, cells were washed 3× with PBS for 3 min (per wash) and incubated for 2 h at room temperature with a secondary antibody. Cells were washed and stained for Hoechst‐33342 as required at room temperature for 5 min and then imaged in the Nikon or confocal microscope (described below). Multifluorescent images across each condition were obtained at 20× magnification. Primary and secondary antibodies against proteins per cell type were used, as detailed in Table S6 (Supporting Information). In monocyte experiments, where required, a combination of antibodies was used. Samples were further analyzed via flow cytometry as detailed elsewhere (Experimental Section, Supporting Information). Excitation and emission wavelengths used for obtaining relevant dot plots are depicted in Table S7 (Supporting Information).

All antibodies were purchased from Abcam (UK), except for primary rabbit anti‐human monoclonal antibody CXCL10 (ThermoFischer Scientific, UK).

### Flow Cytometry

MDA‐MB 231 cells, HUVECs, monocytes, or purified LEVs (the latter derived from MDA‐MB 231 cells as described above) were stained as described above (see also Table S6, Supporting Information). Five hundred microliters of each sample (10 × 10^5^ cells mL^−1^ or 20 × 10^4^ LEVs mL^−1^) were loaded into an Amnis Cell Stream flow cytometer (Luminex, US) and samples were run at a flow rate of 20 µL min^−1^. At least 1000 events per sample and condition were recorded. Excitation and emission wavelengths used for obtaining relevant dot plots are depicted in Table S7 (Supporting Information). For all histograms generated, antibody fluorescence intensity was represented in the *x*‐axis and frequency in the *y*‐axis. As a general approach, unstained samples and single‐color controls were used for setting gating thresholds.

For LEVs internalization studies, pre‐stained cells with a red cell tracker (HUVECs or monocytes or M1 macrophages) were co‐cultured independently with pre‐stained LEVs with a green cell tracker for 16 h. Internalization frequency was defined as the number of double positive events, cells that had internalized LEVs compared to the overall number of cells. Where required, cells were pre‐treated with Cyto‐D, EIPA, or a combination, as described elsewhere, and then LEVs were added. In each case, unstained cells, untreated cells (stained), and LEVs alone (stained) were used as controls for gating the limits for double positive dot plots (cells that had internalized LEVs), and samples were analyzed as above. For relevant experiments, fluorescent beads of 5‐ and 10 µm (microParticles GmbH, Germany) were used.

### RNA Extraction and Real‐Time PCR

HUVECs, THP‐1 monocytes, or THP‐1 derived M1 macrophages were independently co‐cultured with LEVs generated by MDA‐MB 231 cells (as described elsewhere) at ratios of 3 × 10^5^ cells:1 × 10^5^ LEVs. The above cells (3 × 10^5^) were cultured alone as controls. Additionally, 2 × 10^5^ LEVs were cultured alone to investigate their RNA content. Cells or LEVs were centrifuged at 180 × *g* for 5 min or at 10 000 × *g* for 30 min to pellet cells or LEVs, respectively. Then, total RNA was isolated from each sample of cells or LEVs using RNeasy Plus Mini Kit (Qiagen, USA), based on the manufacturer's instructions. The quality, quantity, and purity of the extracted RNA were determined using a Nanophotometer (IMPLEN, USA). One micrograms of the sample was used for reverse transcription, performed via iScript cDNA synthesis kit (BioRad laboratories, UK). Then, 50 ng of cDNA per sample was used and amplified by applying real‐time PCR, using the equipment of Applied Biosystems StepOne Real‐time PCR System (Fischer Scientific, UK). SYBR Green PCR master mix (ThermoFischer Scientific, UK) was used for the final PCR reaction. Relative mRNA expression was extrapolated using the 2^–^ *^ΔΔCt^* method, for all genes and their primer sequences, as listed in Table S8 (Supporting Information).

### Protein Concentration and Gel Electrophoresis

Initially, suspensions of each fraction were washed 1× with chilled (4 °C) PBS and lysed using 300 µL of radioimmunoprecipitation assay (RIPA) lysis buffer (Thermo Fischer Scientific, UK) for 15 min on ice in a plate shaker. All fractions were centrifuged at 18 900 × *g* for 15 min to remove debris, and the supernatant containing the lysate was collected. All other previous centrifugation steps were at 180 ×* g* for 5 min and at 9600 ×* g* for 30 min, for cells and LMPs, respectively. The protein lysates were stored at −80 °C or placed on ice for immediate use.

Protein quantity of MDA‐MB 231 control cells, cells post‐transit (bifurcated capillary devices), and LEVs was measured using Bicinchoninic acid (BCA) assay kit (Life Technologies, UK), per manufacturer's instructions. The absorbance of each sample at 562 nm was analyzed using a Varioskan Flash plate reader (ThermoFischer Scientific, UK).

For qualitative protein analysis, gel electrophoresis was performed. The separating gel was prepared by mixing 1.6 mL PBS, 2 mL Acrylamide/Bis‐acrylamide 30% (v/v) solution, 1.3 mL 1.5 m Tris (Life Technologies, UK), 50 µL 10% (v/v) Sodium Dodecyl Sulfate (Thermo Fischer Scientific), 50 µL 10% (v/v) Ammonium Persulfate (Bio Rad, UK) and 2 µL tetramethylethylenediamine (Bio Rad, UK). The stacking gel was prepared by mixing 1.4 mL PBS, 0.33 mL Acrylamide/Bis‐acrylamide 30% (v/v) solution, 0.25 mL 1.5 m Tris, 20 µL 10% (v/v) Sodium Dodecyl Sulfate, 20 µL 10% (v/v) Ammonium Persulfate, and 2 µL tetramethylethylenediamine. Both gels were left to polymerize at room temperature for 20 min. Both the separating and stacking gels were enclosed in a glass cassette (Bio Rad, UK) and incorporated in the electrophoresis chamber (Bio Rad, UK). Protein samples (250 µg mL^−1^; 15 µL) were mixed with 5 µL Laemmli protein buffer (Bio Rad, UK) and heated for 10 min at 95 °C. Twelve microliters of each sample and protein ladder solution (Bio Rad, UK) were added across the wells of the gel. The electrophoresis chamber was filled with 10% (v/v) Sodium Dodecyl Sulfate, and the samples were run for 120 min at 125 V and 400 A. The gel was then removed carefully from the enclosed glass cassette and stained with 50 mL Coomassie blue (Bio Rad) in a glass sealed container for 1 h at room temperature on a plate shaker (Thermo Fischer Scientific, UK). The staining solution was removed, and the gel was destained using 50 mL of Coomassie brilliant blue R‐250 destaining solution (Bio rad, UK) for 1 h at room temperature on a plate shaker. Images were obtained with a mobile phone (Redmi, China).

### Western Blotting

LEVs purified from MDA‐MB‐231 cells were collected and transferred into a 15 mL tube, then centrifuged at 1500 rpm for 10 min. Following centrifugation, the supernatant was discarded, and the cell pellet was resuspended in 500 µL of 0.1% NaCl solution. The suspension was transferred into a 1.5 mL Eppendorf tube and further centrifuged at 13 000 rpm for 10 min. The resulting supernatant was again discarded. Cell lysis buffer was freshly prepared by mixing 5 mL RIPA buffer (Sigma‐Aldrich, 0000351333), 100 µL PhosSTOP (Roche, 04906837001), and 10 µL protease inhibitor (Sigma‐Aldrich, I3786‐1ML). The cell pellet was resuspended in 20 µL of cell lysis buffer, gently agitated, and incubated on ice for 15 min. After incubation, the suspension was centrifuged at 13 000 rpm for 10 min, and the clarified supernatant (cell lysate) was carefully transferred to a clean Eppendorf tube.

Protein concentration in the lysate was determined using a bicinchoninic acid (BCA) assay. The BCA reagent was prepared by combining 49 mL Reagent A (Thermo Scientific, 23228) with 1 mL Reagent B (Thermo Scientific, 1859078). Lysate samples were diluted at a ratio of 1:20 (1.5 µL cell lysate with 28.5 µL cell lysis buffer) and maintained on ice. A protein standard curve was generated using bovine serum albumin (BSA) at concentrations of 1000, 750, 500, 250, 125, 25, and 0 µg mL^−1^, diluted in cell lysis buffer. Standards and diluted lysate samples (25 µL each) were loaded into a 96‐well plate, followed by the addition of 200 µL BCA reagent to each well. The plate was shaken gently for 30 s, incubated at 37 °C for 30 min, and absorbance was measured at 570 nm using a Tecan plate reader. Based on BCA results, lysate volumes containing 30 µg of total protein were calculated. Samples were prepared to a final volume of 50 µL comprising 12.5 µL of 4X LDS buffer (Invitrogen, NP0007), Five microliters of 10× reducing agent (Invitrogen, NP0009), the calculated lysate volume, and cell lysis buffer.

Electrophoresis running buffer was prepared by combining 950 mL ultrapure water with 50 mL NuPAGE SDS MOPS running buffer (20×). Samples were loaded onto a 4–12% Bis‐Tris gel (NP0321BOX). The gel cassette was initially placed in a shallow bath of running buffer to remove the tape and comb, ensuring no gel residues obstructed the wells. The gel cassette was then positioned in a mini gel tank (ThermoScientific, A25977) filled with running buffer, with wells oriented forward. Samples were vortexed briefly, heated at 90 °C for 3 min to denature proteins, then cooled to room temperature before loading onto the gel. PageRuler Ladder (5 µL; ThermoScientific, 26619) was loaded into the first lane for molecular weight referencing. Subsequently, 20 µL of the prepared sample was loaded into the individual well, and electrophoresis was performed at 140 V for 1 h.

Post‐electrophoresis, the gel cassette was opened in a shallow bath of running buffer, and the gel was gently released using a scraper. The gel was transferred to an iBlot cassette, air bubbles were carefully removed using a roller. The cassette was closed and transferred to an iBlot 3 Western Blot Transfer System, run using the broad molecular weight range setting. Successful protein transfer was confirmed by visible ladder transfer onto the membrane. The membrane was briefly rinsed in tris‐buffered saline (TBS) and blocked in a solution consisting of 5 mL Licor Intercept Blocking Buffer (Licor, 927–60001) and 5 mL ultrapure water for 1 h at room temperature on a rocker.

Primary antibody incubation was performed overnight at 4 °C to detect HSP90 and CD81 as previously, using a solution containing 5 mL Licor Intercept Blocking Buffer, 5 mL ultrapure water, and 10 µL Tween‐20 (Sigma‐Aldrich, P7949). Vinculin‐ (E1E9V) XP (Cell Signal Technologies #13901) was used as dye loading control at 1:1000. After incubation, membranes were washed three times in TBST (TBS containing 0.1% Tween‐20) for 10 min each at room temperature. Secondary antibody incubation followed the same protocol as previously, with subsequent washes in TBST performed three times, each for 5 min. Finally, the membranes were scanned using the Li‐cor Odyssey XF imaging system, detecting the fluorescent signals at wavelengths corresponding to the secondary antibodies used.

### Sample Preparation for Mass Spectrometry and Proteomics

Control cells (500 000), cells post‐shedding, and purified LEVs were prepared as previously. Cells and LEVs were pelleted at 180 × *g* for 5 min or at 10 000 × *g* for 30 min, respectively, to remove media and washed 1× with cold PBS for 5 min. Pelleted samples were lysed by adding 200 µL of 8 m freshly prepared urea (SIGMA‐ALDRICH, St Louis, MO, USA). This lysis step was performed on ice for 20 min with brief vortexing throughout. Samples were then transferred to −80 °C for storage. Protein quantification was performed using Pierce Protein Assay Kit (Thermo Scientific, Rockford, IL, USA) per the manufacturer instructions.

A total of 0.4 µg µL^−1^ protein (150 µL total) was used per sample for sample processing and digestion. Reduction of disulfide bonds was carried out by adding Dithiothreitol (DTT, SIGMA‐ALDRICH, St Louis, MO, USA) at a working concentration of 10 mm and incubation for 40 min and 50 °C. Samples were then alkylated using a 50 mm working concentration of iodacetamide (IAA, SIGMA‐ALDRICH, St Louis, MO, USA) and incubation for 30 min at room temperature in the dark. Samples were then diluted with 100 mm ammonium bicarbonate (ABC) to achieve a final urea concentration of 2 m. This was followed by trypsin digestion, where trypsin (Pierce Trypsin Protease MS‐grade, Thermo Scientific, UK) was reconstituted in 100 mm ABC and was used at a concentration of 1 µg per 100 µg of total protein. Sample digestion was carried out for 18 h at 37 °C. Trypsin digestion was then quenched with 0.5% (v/v) Trifluoroacetic acid (TFA, Fisher Scientific, Leicestershire, UK) and samples were desalted using SepPak C18 Plus cartridges (Waters, Milford, MA, USA). Finally, peptides were dried using a speedvac concentrator (Savant SC250EXP SpeedVac, Thermo Scientific) and stored at −80 C.

For mass spectrometry analysis, peptides were reconstituted in 0.1% trifluoracetic acid (TFA) at a concentration of 200 ng µL^−1^ for liquid chromatography–mass spectrometry (LC–MS) Data Independent Acquisition (DIA) analysis. DIA analysis was performed on an UltiMate 3000 system (Thermo) coupled with the Orbitrap Ascend Mass Spectrometer (Thermo) using a 25 cm capillary column (Waters, nanoE MZ PST BEH130 C18, 1.7, 75 × 250 mm). One microgram of peptide was analysed per sample. DIA was performed over a 100 min gradient, 5%‐35% mobile phase B composed of 80% acetonitrile, 0.1% formic acid. MS1 spectra were collected with mass resolution of 60 K in the m/z range of 380–985, with Maximum Injection Time 100 ms and automatic gain control (AGC) 4×105. DIA MS2 spectra were collected with higher energy collisional dissociation (HCD) fragmentation collision energy (CE) 32%, orbitrap resolution 15 K with isolation window 10 m/z and 1 m/z overlap, Maximum Injection Time 40 ms and AGC 1 × 105. Raw data were processed in the DIA‐NN software version 1.8.1^[^ ^44^ ^]^ for protein identification and quantification using a fasta file containing reviewed UniProt human proteins.^[^ ^45^ ^]^ Carbamidomethylation of C and oxidation of M were defined as fixed and variable modifications, respectively. Retention time (RT)‐dependent cross‐run normalization was selected with a match between run (MBR) enabled, and proteins were filtered at a 1% False discovery rate. Gene identifier names (Gene IDs) were filtered to only include proteins identified in at least 75% (2/3) of samples. Proteins identified in LEVs were searched against gene sets from the Reactome database (https://reactome.org/). The top 15 gene sets were ranked based on the number of overlapping gene IDs with LEVs. LEVs’ protein annotations relevant to protein secretion molecular functions, as analyzed by pathway analysis, were extracted from the database.

### LEV Uptake under Flow

For this series of experiments, a microfluidic device (length: 50 mm, width: 1 mm), previously established in the lab was used.^[^ ^46^ ^]^ The inlet of a microfluidic device was connected to the outlet of a 12 V adjustable peristaltic dosing pump (Amazon, UK, G628‐1‐2) via 50 cm long tubing of 2 mm diameter (Qosina, US). The outlet of the microfluidic device was connected with a 50 cm long tubing of 2 mm diameter, and the free end was inserted in a 50 mL falcon tube that was prefilled with 10 mL of media. A third piece of 50 cm long tubing of 2 mm diameter was inserted in the inlet of the peristaltic pump, and its free end was also inserted in the same 50 mL falcon tube. Purified and pre‐stained LEVs (10 × 10^4^; CMFDA green cell tracker) were added in the falcon tube either with 30 × 10^4^ pre‐stained THP‐1 monocytes (CMPTX red cell tracker) or with 30 × 10^4^ pre‐stained THP‐1 derived M1 macrophages (CMTPX red cell tracker) separately. In each experiment, the peristaltic pump was turned on for continuous circulation of cells and LEVs. After 16 h of co‐culture under flow, the media was collected, centrifuged at 180 × *g*, and the pellet was resuspended in fresh media. Five hundred microliters of each sample was collected, and LEV internalization was compared using flow cytometry as described above.^[^ ^47^ ^]^

### RNA Silencing

10^5^ MDA‐MB‐231 tumor cells were grown in each well of a 24‐well plate for a total of 2 × 10^6^ cells. TGFb was knocked down by following the siRNA transfection protocol using DharmaFECT (Horizon Discovery) transfection according to the manufacturer recommended protocols. Cells were cultured for an additional 48 h, and the media was changed every day. Then, cells were harvested and used to generate and count LEVs as described previously.

### 2D HUVEC Cell and Dextran Permeability Assays

HUVECs (15 × 10^3^) were cocultured in wells of a 96 well plate with 5 × 10^3^ purified LEVs derived from MDA‐MB 231 cells or 5 × 10^3^ purified LEVs derived from siTGFb‐transfected MDA‐MB 231 cells as described above. Equal numbers of HUVES but cultured without LEVs were used as negative controls. HUVECs were pre‐stained with a CMFDA green cell tracker. At 0 h, both fluorescent and brightfield images were obtained at 10× and 20× magnification. After 30 h, similar images were obtained again to characterize the extent of HUVEC monolayer disruption. In other experiments, HUVECs were stained with VE‐cad and Hoechst instead of CDFMA cell tracker as described above. Cell‐free versus cell‐containing areas were extrapolated using ImageJ.

HUVEC monolayer disruption was further characterized by performing a dextran permeability assay. Millicell hanging cell culture inserts of either 0.4 or 8 µm pores (Merck, UK) were transferred in wells of a 24‐well plate. The well was filled with 900 µl around the cell culture insert, and 200 µL of HUVECs (75 × 10^3^ cells mL^−1^) were then transferred inside the cell culture inserts and left for a day to attach and form the monolayer. Then, HUVECs were treated with 5 × 10^3^ purified LEVs derived from MDA‐MB 231 cells (as described elsewhere) for 30 h. Then, 100 µL of fresh media was added to 0.4 or 8 µm, containing either 10 000 molecular weight Dextran Alexa Fluor 647 (ThermoFischer Scientific, UK) or 10 × 10^4^ cells mL^−1^ pre‐stained MDA‐MB 231 cells (green cell tracker), respectively, for 24 h. Hundred microliters of media were collected from the bottom of the well at 24 h, and fluorescence was measured using a Varioskan Flash plate reader (ThermoFischer Scientific, UK) at 650/668 nm values of peak excitation/emission. The fluorescence values obtained at 24 h were normalized initially based on blank samples (cell‐free media) and baseline subtracted using fluorescence at 0 h.

### 3D Microfluidic Culture of Endothelial Networks

Commercial microfluidic devices (IdenTx, AIM Biotech, Singapore), consisting of three parallel microchannel segments separated by pillars were used. Ten microliters of Matrigel solution (1 mg mL^−1^, Corning, UK), 5 µL of fibrinogen (6 mg mL^−1^, Sigma, UK), and 5 µL of thrombin (4 units mL^−1^, Sigma, UK) were mixed. Then, 50 × 10^4^ HUVECs were resuspended in 20 µL of the mixed solution and immediately injected into the central microchannel segment. All the cell seeding procedures were performed with devices placed on ice. Seeded chips were then incubated at 37 °C for 30 min to solidify the gel, before filling the side channels with pre‐warmed media, as above. To create pressure‐driven flow in the chip, one of the adjacent media channels was filled with 200 µL of the media, and the other channel was filled with 100 µL of the media. The chips were kept in the incubator (37 °C/5% CO_2_) for 5‐7 days to form 3D endothelial networks, and media was changed every 24 h. LEVs, generated and isolated as previously from MDA‐MB 231 cells, were enumerated via flow cytometry. The generated LEVs were incubated with the developed 3D endothelial networks under pressure‐driven flow for 24 h at a cell: LEV ratio of 3:1. Then, 3D endothelial networks were fixed and stained for VE‐cad, as described previously. Fluorescent images were acquired using a confocal microscope, and cell‐free area and fluorescence intensity were calculated with ImageJ (3 biological replicates and 3 technical replicates).

Perfusion studies were conducted by diluting 20 µL of 1 µm fluorescent carboxylate‐modified microspheres (Fluospheres, Invitrogen) 1:100 in media. This solution was injected into one side channel of the endothelial network‐laden IdenTx devices to initiate flow by hydrostatic pressure. Confocal microscopy was employed as previously to capture images of the microparticles and the networks. Quantitative analysis of microparticle colocalization with the VE‐cadherin stained endothelial networks was performed using ImageJ software (3 biological replicates and 3 technical replicates).

### Monocyte Differentiation

CM was collected from untreated HUVECs or HUVECs cocultured with LEVs (derived from MDA‐MB 231 cells as above), as described above. THP‐1 monocytes (15 × 10^3^) were transferred into wells of a 96‐well plate, and 100% of 200 µL of CM from above conditions was added for 30 h. Additional experimental conditions included the addition of either 5 × 10^3^ LEVs or CM collected from LEVs on monocytes for 30 h. Untreated monocytes were used as a negative control. After 30 h, media was removed and monocytes were stained via immunocytochemistry for CD206 and TNF‐a, as described above. Multifluorescent and brightfield images were obtained at 20× magnification, using the confocal microscope. Alternatively, 10 × 10^4^ THP‐1 monocytes were added in the wells of a 24‐well plate, and all the above experimental conditions were repeated. Purified LEVs (33 × 10^3^) either from MDA‐MB231 cells or siTGFb‐transfected MDA MB‐231 cells were co‐cultured with monocytes, as above. Five hundred microliters from each sample were collected and analyzed via flow cytometry as described above. The frequency of CD206+ events was calculated to estimate the polarization of monocytes toward M2 macrophages.

### Monocyte Adhesion

Monocytes (15 × 10^3^) were stained with a CMTPX red Cell Tracker and were then transferred in wells of a 96‐well plate. Then, monocytes were treated independently with 5 × 10^3^ purified LEVs (derived from MDA‐MB 231 cells as above), with 100% CM collected from LEVs and 100% CM collected from MDA‐MB 231 cells (as described above) for 30 h. Untreated monocytes were used as a negative control. Fluorescent images of each well were obtained before and after media removal (removing non‐adhered monocytes), and the percentage of remaining adhered cells was calculated.

Alternatively, 15 × 10^3^ HUVECs were added in wells of a 96‐well plate and left to grow for 1 day. In the meantime, 30 × 10^3^ THP‐1 monocytes were cultured in wells of a 48‐well plate, left to grow for 1 day and then stained with a CMFDA green cell tracker. HUVECs or THP‐1 monocytes were treated with 5 × 10^3^ or 10 × 10^3^ purified LEVs (derived from MDA‐MB 231 cells as above) for 30 h, respectively. Negative control of both untreated HUVECs and untreated THP‐1 monocytes was used. After 30 h, media was removed from both HUVECs and monocytes to remove non‐internalized LEVs, and four experimental conditions were performed: a) untreated THP‐1 monocytes were added to untreated HUVECs monolayers, b) untreated THP‐1 monocytes were added to LEV‐pre‐treated HUVECs monolayers, c) LEV‐pre‐treated THP‐1 monocytes were added to untreated HUVECs monolayers, and d) LEV‐pre‐treated monocytes were added to LEV‐pre‐treated HUVECs monolayers. Using a Varioskan flash plate reader, the green fluorescence of monocytes at 0 h was measured at 492/517 nm values of peak excitation/emission. After 4 h of co‐culture, the media was removed from each well, and fresh media was added. Fluorescence of monocytes was measured by a plate reader as described above. Cell‐free media was used for normalization, and the percentage of remaining fluorescence after media removal (removing non‐adhered monocytes) was estimated.

### Monocyte Proliferation Assay

Monocytes (12 × 10^3^) were added in each well of a 96‐well plate and left to grow for 1 day. Then, they were treated independently with 4 × 10^3^ purified LEVs (derived from MDA‐MB 231 cells as above) or 100% CM collected from LEVs (as described above) for altogether 48 h. Untreated monocytes were used as a negative control. To evaluate monocyte proliferation, the MTT cell proliferation assay kit (Abcam, UK) was used, per the manufacturer's instructions. The absorbance at 590 nm of each well was read using a Varioskan flash plate reader and normalized as described elsewhere.

### Graphs and Statistical Analyses

All data were presented as mean ± standard deviation (SD). Unless stated otherwise, all experiments were performed independently at least three times (*n* = 3) for each experimental condition, shown for each experiment in the relevant caption. Data were plotted graphically and analyzed using GraphPad Prism software (version 9.0). Significance analyses were done by applying either two‐tailed Student's *t*‐test or one‐way analysis of variance (ANOVA ‐Turkey's multiple comparisons) between experimental groups (95% CI). *P*‐values ≥ 0.05 were considered as not significant. *P*‐values below this value were considered as significant (0.01 to 0.05, single asterisk*), very significant (0.001 to 0.01, double asterisk **), extremely significant (0.0001 to 0.001, triple asterisk *** or < 0.0001, quadruple asterisk ****). Illustration was prepared using Biorender.

## Conflict of Interest

The authors declare no conflict of interest.

## Author Contributions

A.V. and S.H.A. conceptualized the project. A.V. designed all experiments, fabricated microfluidic devices and ran all microfluidic experiments, developed methodologies on LEVs generation and purification, characterized LEVs, performed LEVs co‐culture experiments, analyzed data and conducted statistical analyses. B.C. conducted western blotting and G.S. assisted with Western blotting and conducted centrifugal force experiments. S.B.S. performed the 3D vascular network formation experiments and analyzed data. S.A. conducted proteomics experiments and S.A. and P.H. conducted proteomics analysis. K.S. and M.C. processed patient and clinical samples. F.B. supervised clinical sample collection. K.S., S.B.S., K.L.S., and C.D. coordinated CDX experiments. K.S., K.L.S., and C.D. coordinated primary CTC experiments. A.V. performed primary CTC experiments on microfluidics and identified fragments in patient samples. T.M.P. performed cryoEM experiments and analyzed data. M.A., A.Z., and D.V. assisted with macrophage, monocyte and endothelial experiments, respectively. A.C. assisted with cluster experiments. S.H.A. conducted computational fluid dynamics and supervised the overall project direction. A.V. and S.H.A. wrote the manuscript with edits from all authors.

## Supporting information



Supporting Information

Supplemental Movie 1

Supplemental Movie 2

Supplemental Movie 3

Supplemental Movie 4

Supplemental Movie 5

Supplemental Movie 6

Supplemental Movie 7

Supplemental Movie 8

Supplemental Table 1

## Data Availability

Data that support the findings of this study are openly available in ProteomeXchange at www.proteomexchange.org, reference number PXD050444.
